# The interplay between haptic guidance and personality traits in robotic-assisted motor learning

**DOI:** 10.1186/s12984-025-01709-6

**Published:** 2025-11-12

**Authors:** Alberto Garzás-Villar, Caspar Boersma, Alexis Derumigny, J. Micah Prendergast, Arkady Zgonnikov, Jane Murray Cramm, Laura Marchal-Crespo

**Affiliations:** 1 Department of Cognitive Robotics, Delft University of Technology, Mekelweg, Delft, 2628 CD Zuid Holland Netherlands; 2 Department of Applied Mathematics, Delft University of Technology, Mekelweg, Delft, 2628 CD Zuid Holland Netherlands; 3 Department of Socio-Medical Sciences, Erasmus School of Health Policy & Management, Erasmus University Rotterdam, Burgemeester Oudlaan, Rotterdam, 3062 PA Zuid Holland Netherlands; 4 Department of Rehabilitation Medicine, Erasmus MC, Dr. Molewaterplein, Rotterdam, 3015 GD Zuid Holland Netherlands

**Keywords:** Personality traits, Motor learning, Robotic-assisted rehabilitation, Haptic guidance, Personalization

## Abstract

**Background:**

Robotic devices have shown promise in supporting motor (re)learning. However, there is a limited understanding of how personality traits influence the effectiveness of robot-aided training strategies.

**Methods:**

We conducted a motor learning experiment with 40 unimpaired participants who trained to control a virtual pendulum using a robotic haptic device. Before the experiment, we assessed personality traits including the perceived control over life events (Locus of Control), the tendency to turn challenges into engaging activities (Transform of Challenge), and other subscales from Autotelic and Hexad gaming style questionnaires. Participants were divided into two groups, one receiving haptic guidance during training and a second one without assistance. Short- and long-term retention was assessed, and relationships between personality traits, performance metrics, and human-robot interaction metrics were analyzed.

**Results:**

Participants with high Transform of Challenge or external Locus of Control characteristics who received physical guidance during training reduced the human-robot interaction forces to a lesser extent compared to the ones who did not receive guidance. Additionally, participants with a high Free Spirit gaming style showed greater sensitivity to how their perception of the guidance affected their performance during the retention phases.

**Conclusion:**

Our findings suggest that autotelic personality, Locus of Control, and gaming style modulate motor learning outcomes during robotic-assisted training, affecting both performance and human-robot interaction metrics. This highlights the potential of integrating personality-based adaptations in robot-aided rehabilitation protocols to enhance performance and motor (re)learning. Future works should explore the relationship between personality traits and psychological states (e.g., perceived difficulty, attention) across diverse tasks and guidance methods in clinical populations.

## Introduction

Damage of the central nervous system, e.g., after stroke, often results in motor impairments that require re-learning of lost motor skills [1]. Technological advances, such as robotics and virtual reality (VR), have shown promise in supporting motor (re)learning by providing augmented feedback, e.g., in the form of visual, auditory, haptic, or their multimodal information combinations [2]. Among these, the provision of haptic feedback is one of the most popular approaches in robot-aided training. Haptic feedback has shown potential in positively influencing motor learning when combined with other feedback forms [2, 3]. In particular, haptic guidance — where robots physically assist users in achieving correct movements [4]—has been shown to enhance motor learning, especially in those initially less skilled or in the early stages of stroke neurorehabilitation [3, 5]. There is also evidence that robotic training strategies that challenge users, e.g., by amplifying errors, may benefit more skilled users [6, 7].

Despite previous research pointing towards individual differences in robot-assisted motor learning, there is a lack of understanding of how to optimally select haptic training strategies for each individual trainee. Different training strategies affect not only motor performance during training but also trainees’ motivation [3, 8–10], which in turn influences motor learning [11]. To date, determining the most suitable training approach for each individual is hindered by insufficient knowledge of the intrinsic human factors that play a role in the effectiveness of robot-aided training [12, 13].

Factors that might differentiate trainees are multifaceted, ranging from variations in individual characteristics — e.g., physiological or psychological differences [14, 15] — to environmental and social factors [16, 17]. Adapting robotic assistance to trainees’ physiological measures and physical capacities has shown benefits in motor learning and neurorehabilitation [18, 19]. Yet, there is increasing recognition of the potential value of incorporating other characteristics, such as personality traits, personal preferences, and psychological states to provide more personalized robotic training programs [20–24].

While psychological states — e.g., motivation and engagement — are dynamic and can change depending on circumstances [14, 25], personality traits are stable characteristics that shape how people consistently think, feel, and behave over time [15]. Since personality traits are expected to be stable, such information could reduce the need for *in vivo* data collection. Personality traits have been shown to be relevant in different areas such as sports [26], employee productivity [27], online gaming [28], or academic learning [29]. However, despite the potential relationship between personality traits and motor learning, their interplay with robotic-aided training has not been systematically investigated.

To address this gap, we performed a parallel design experiment involving 40 unimpaired participants who learned to control a virtual pendulum with one internal degree of freedom to hit upcoming targets with the pendulum mass in a VR-based game. Participants could control the pendulum by moving the pendulum’s pivoting point through a haptic device. Participants could feel the pendulum dynamics through the haptic device. During training, the device supported half of the participants (*Experimental* group) through haptic guidance. This group was compared against a *Control* group that did not receive this guidance. Using questionnaires, we measured two personality traits and two gaming styles for each participant.

- **Autotelic personality**. According to the challenge point framework [30], optimal learning occurs when learners face challenges that are neither too easy nor overwhelming, facilitating task engagement. Therefore, personality traits that influence how individuals perceive and approach challenges may be crucial during robotic-assisted motor learning. The autotelic personality trait gives information about the disposition to seek challenges or enter the “flow” state [31] and has already shown its potential to predict engagement in sports settings [32]. In this study, we assessed autotelic personality using the *Transform of Challenge* and *Transform of Boredom* subscales from the Autotelic Personality Questionnaire [33]. These subscales reflect an individual’s tendency to transform “challenging” or “boring” situations, respectively, into personally motivating ones. These subscales might be relevant when interacting with robotic devices, as they might interact with how the robotic assistance influences participants’ engagement in tasks whose functional difficulty — i.e., how challenging the task is perceived — can be modulated by physically assisting the movement [9].
- **Locus of Control** (LOC) is related to individuals’ sense of control over their actions and outcomes [34]. Autonomy and perceived control can be of particular interest when interacting with robotic devices that provide physical assistance [20]. Previous research indicated that participants’ LOC (internal vs. external) can impact how they react to external feedback [35], as well as their interaction with robotic devices [36]. For instance, in this last work, Acharya et al. found that LOC was correlated with how participants behaved under different types of assistance in a robotic teleoperation task.
- **Gaming styles**. Gamification can motivate trainees [37] but can be intricate as it intertwines with personal preferences and characteristics that can either boost or hinder engagement [38]. An established method for capturing this is to classify the user’s gaming style, which characterizes users based on their interaction preferences within game-like environments [39]. Though not a personality trait, gaming style can be potentially used to assess the trainee’s personality profile [40]. Here, we assessed participants according to two gaming styles: *Achiever* — reflecting a preference for challenges — and *Free Spirit* — reflecting a tendency for exploration [41]. These gaming styles may influence how users respond to structured interactions like haptic guidance, which can be perceived as a game element that limits free exploration or directly impacts task difficulty.

The experimental protocol was divided into two sessions, 1–3 days apart. The first session included a personality traits assessment, a baseline to determine participants’ initial skill level, a training phase, and a short-term retention (STR) evaluation. The second session included a long-term assessment of motor learning. During the baseline and the two retention phases, the participants performed two transfer tasks to asses the generalization of the acquired skill [3, 42]. The transfer tasks were similar to the trained task (referred to as the *main task*) but included slight design variations. In the *position transfer task*, the targets were randomly re-located from the trained *main tasks* to evaluate the participants’ ability to adapt to unfamiliar spatial patterns. The second transfer task, referred to as *dynamics transfer task*, introduced variations in the pendulum dynamics, i.e., the pendulum rod length was reduced by 70%, while the target positions were the same as in the *main task*.

In this paper, we present results on the analysis of the relationship between personality traits, haptic guidance, human-robot interaction forces, and participants’ motor learning by testing the following hypotheses.

- **H1** **Personality traits and presence of haptic guidance influence how task performance and human-robot interaction force change from baseline to retention.**
  - *H1.1* *Haptic guidance:* Participants were anticipated to perform worse during short-term retention if they were allocated to the *Experimental* group, compared to those allocated to the *Control* group, due to reliance on the haptic guidance.
  - *H1.2* *High Transform of Challenge participants:* For participants allocated to the *Experimental* group, performance during short-term retention was expected to decline even more if they had high levels of the Transform of Challenge trait. This is based on findings in [43], where participants with high Transform of Challenge showed worse performance when receiving guidance compared to not receiving guidance during the training phase.
  - *H1.3* *High Achievers:* Participants with high levels of Achiever gaming style were expected to outperform average participants. This is irrespective of the training group they were allocated to and based on their improved performance w.r.t. average participants during the training phase [43].
  - *H1.4* *Human-robot interaction force and Free Spirits:* Participants with high Free Spirit gaming style in the *Control* group were expected to exhibit higher interaction forces with the haptic device during short- and long-term retention compared to an average participant, which they already showed during the training phase [43].
  - *H1.5* *Human-robot interaction force and Locus of Control:* Participants with high LOC scores (external LOC) allocated in the *Experimental* group were expected to exhibit greater interaction forces from baseline to STR compared to a participant with low LOC scores (internal LOC), as they already showed this trend during the training phase [43].
- **H2** **Different movement patterns (target positions) and pendulum dynamics affect task performance and human-robot interaction force and are influenced by personality traits.**
  - *H2.1* *Transfer task with different target positions*: During baseline, participants were expected to perform worse in terms of both performance and interaction force metrics in the *position transfer task* compared to the main task. This is due to the potential added challenge of adjusting to new, less familiar target positions. However, we hypothesized this effect would diminish during the retention phases due to learning after the training.
  - *H2.2* *Transfer task with different pendulum dynamics*: Participants were expected to perform better during this transfer task compared to the main task in all phases. In this transfer task, as the pendulum rod was shortened, the pendulum’s natural frequency increased. In [44], Özen et al. showed how operating the system far from the pendulum’s natural frequency was correlated with higher control. We hypothesized that keeping the same target positions within walls, but increasing the pendulum’s natural frequency, would facilitate controlling it.
  - *H2.3* *Transfer task and Transform of Challenge*: Participants with high Transform of Challenge were expected to improve the performance metric in a smaller amount than the average participant in those tasks potentially less challenging — i.e., the pendulum is easier to control (*H2.2*) — and outperform average participants in those involving new and unexpected target positions (*H2.1*).
- **H3** **Subjective perception of human-robot interaction correlates with task performance metrics and personality traits.**
  - *H3.1* *Interaction perception and performance:* Positive responses to the robot interaction perception questions (e.g., perceiving the robot as permitting, helpful, or non-frustrating) were expected to be associated with better performance improvements after training.
  - *H3.2* *Interaction perception and personality traits:* We also expected that the subjective perception of the haptic guidance would interact with personality traits and affect the performance and human-robot interaction force decrease after training.

We did not include hypotheses specifically addressing the relationship between personality traits, haptic guidance, and motor performance during training, as this specific experimental phase was analyzed in a prior study published in a conference proceeding (see [43]).

## Methods

### Experimental setup

**Fig. 1:**
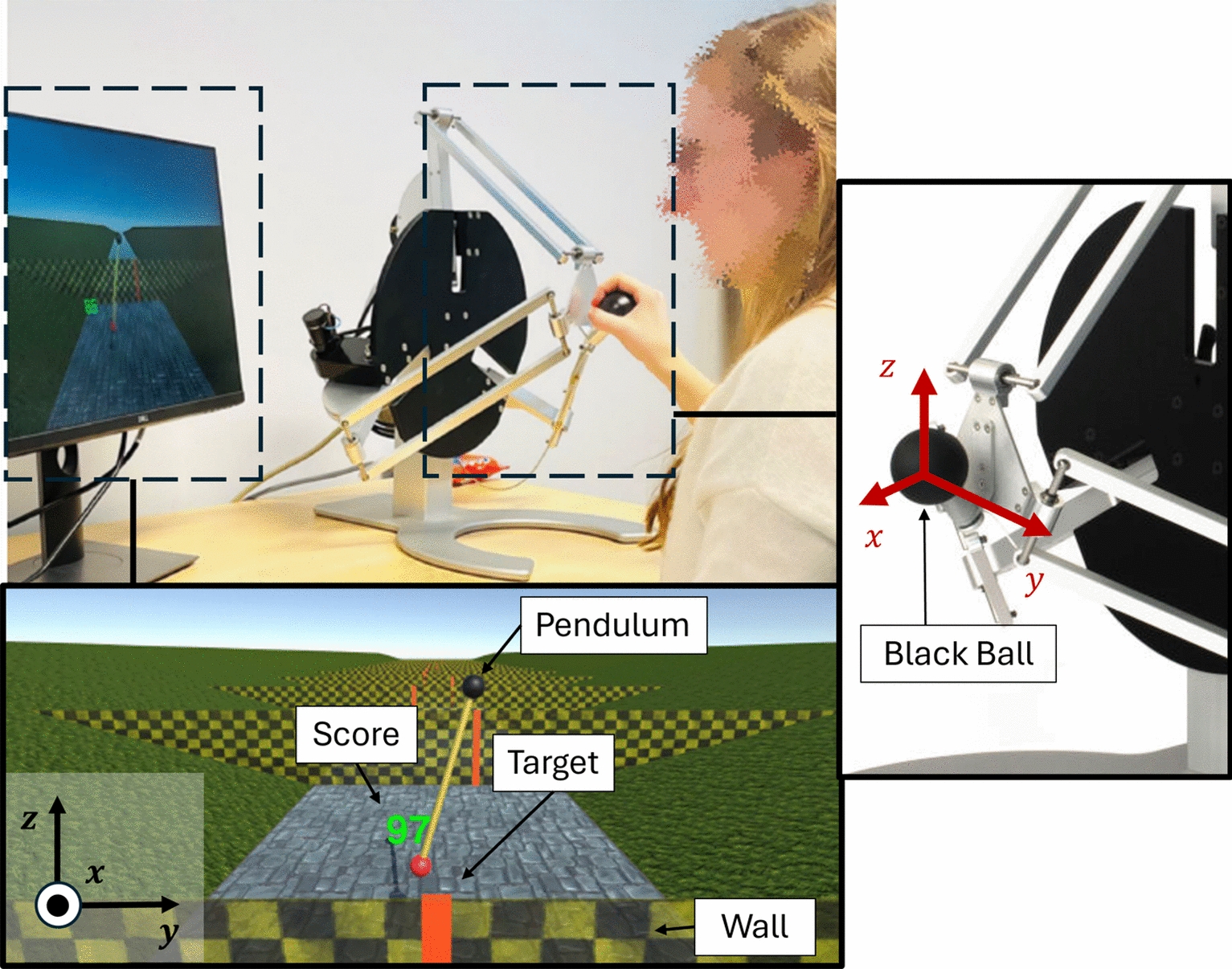
The experimental setup (up left) consists of the screen and the $Delta.3$ (Force Dimension, Switzerland). Down left: Game screenshot with the pendulum, walls in black and yellow, targets as vertical red lines, and the score in green numbers. Right: The device could be controlled by holding the black ball attached to the robot end-effector

The experimental setup included a $Delta.3$ haptic robot (Force Dimension, Switzerland) placed on a desk next to a display monitor (Fig. 1). The device is capable of measuring positions and providing forces up to 20.0 N in the three translational directions (*x*, *y*, and *z* axes, Fig. 1). The device control was implemented in C++, operating at 4 kHz. Motion data was recorded at 1.67 kHz.

### The pendulum game

**Fig. 2:**
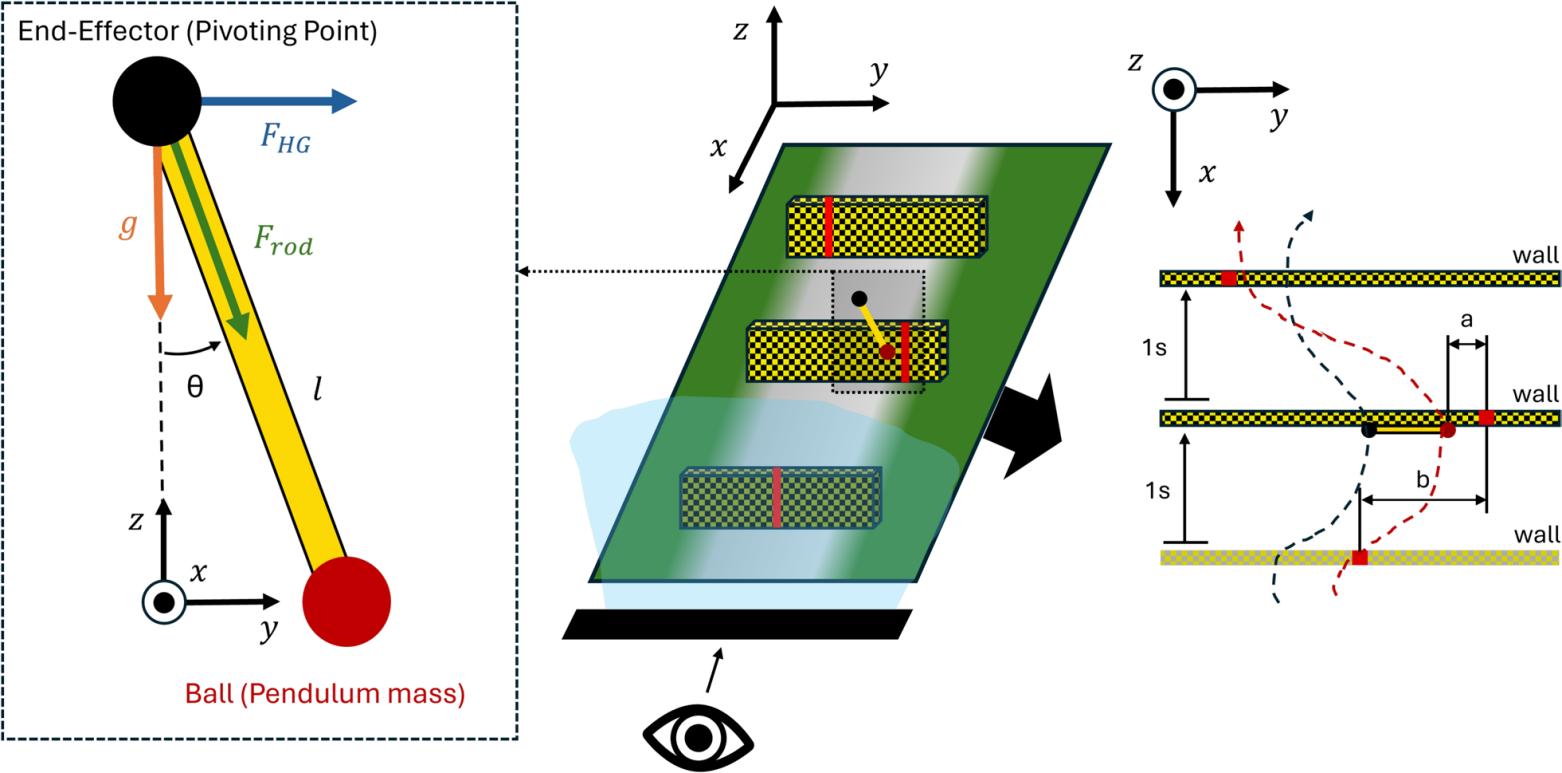
Left: Front view of the pendulum with forces applied on the pivoting point. $F_{HG}$ represents the force from the haptic guidance while $F_{rod}$ is the force from the pendulum dynamics. Center: 3D representation of the game. Right: Top view representation of the game with exemplary trajectories of the pivoting point and the pendulum mass, in black and red dashed lines respectively. The red lines within the walls represent the targets. The variable *b* represents the target position with respect to the centerline, while the variable *a* represents the absolute error between the pendulum mass and the target

The game, inspired by the work of [44] and created in Unity 3D (Unity Technologies, USA), consisted of controlling a virtual pendulum to hit moving targets approaching the participant. The pendulum consisted of a black ball (pivoting point) and a red ball (pendulum mass), with a rigid link connecting both balls, as shown in Figs. 1 and 2. The pendulum’s pivoting point could be moved horizontally and vertically (*y* and *z* axis in Fig. 2) by displacing the haptic device’s end-effector (black ball in Fig. 1 Right; 1:1 movement mapping). The pendulum could only swing in the vertical plane (*y*-*z*), and therefore, movements of the haptic device in the *x*-direction were not mapped to the pendulum.

The task consisted of hitting vertical targets with the pendulum mass. The targets were located on walls approaching the participants in the *x* direction, i.e., perpendicular to the screen plane. The walls were spaced by 1 m and their speed was set to 1 m/s. The targets could appear in three different positions: the center point of the wall or ± 0.12 m to the right/left. The target’s width was 0.02 m, and the pendulum ball and pivoting point diameter were set to 0.03 m.

By moving the pendulum pivoting point through the device end-effector, participants influenced the swing of the pendulum, which behaved according to the equation of motion of a simple pendulum:

1 $$\begin{aligned} \ddot{\theta }=-\frac{1}{l}((\ddot{z}+g)\sin {\theta } + \ddot{y}\cos {\theta })- \frac{c}{ml^2}\dot{\theta }, \end{aligned}$$

where *y* and *z* are the horizontal and vertical coordinates of the robot end-effector position and $\ddot{\theta }$ the angular acceleration of the pendulum’s internal degree of freedom (DoF). Since the internal DoF was located at the pendulum’s pivoting point, $\theta$ was defined relative to the pendulum rod, as illustrated in Fig. 2. The robot’s coordinates were referenced with respect to its initial position after calibration, similar to the one shown in Fig. 1*right*. The pendulum mass was set to $m = 0.6$ kg, the rod length to $l = 0.25$ m, gravity to $g = 3.24$ m/$s^2$, and the constant $c = 3.00e^{-6}$ N$\cdot$s/rad. These parameters were adjusted and chosen in order to minimize passive stabilization of the pendulum and maintain task difficulty.

As the pendulum crossed a wall, a score based on the absolute distance of the pendulum’s mass to the center of the target in the *y*-direction (|*Error*|) was briefly displayed for 0.5 s to provide feedback regarding participants’ performance. The score ranged between 0 and 100 and was calculated as:

2 $$\begin{aligned} Score = {\left\{ \begin{array}{ll} 0 & \text {if } |Error| \ge 0.2\,m, \\ 100-500\cdot |Error| & \text {if } |Error| < 0.2\,m. \end{array}\right. } \end{aligned}$$

Each phase of the experiment was organized into wall sets, with 20 walls presented per set (see the Study protocol Section). A final score, based on the average of all 20 scores, was shown at the end of each set to inform participants of their overall performance in that set.

### Haptic rendering and haptic guidance

To enhance the ecological validity of the task — ensuring the experimental conditions closely replicate real-world scenarios — we incorporated haptic rendering throughout the whole experiment, i.e., the provision of the forces originating from the pendulum dynamics on the device end-effector. Participants could feel the pendulum force dynamics ($F_{rod}$), calculated as:

3 $$\begin{aligned} F_{rod}=m((\ddot{z}+g)\cos {\theta }-\ddot{y}\sin {\theta }+\dot{\theta }^2l), \end{aligned}$$

using the same constants as in Eq. (1).

Participants allocated to the *Experimental* group were also provided with haptic guidance during training to physically assist them in the target-hitting task. This was achieved by first calculating an optimal end-effector trajectory between the pendulum state at the moment of target wall collision and the target at the following wall, and then enforcing this trajectory using a Proportional-Derivative (PD) controller.

The optimal end-effector trajectory was calculated every time the pendulum hit a target wall using the ACADO toolkit [45]. The cost function included terms to maximize accuracy (i.e., minimize the distance between the pendulum ball and the next target’s centerline), maximize the pendulum stabilization (i.e., penalizing the velocity components of the pendulum ball), and minimize end-effector acceleration based on the current state of the pendulum, as described in the Appendix B.

The PD controller aimed to minimize the distance between the end-effector and the reference trajectory in the *y*-direction at each time point by applying a guiding force $F_{HG}$ at the end-effector. We only provided guidance in the *y*-axis as it was sufficient to achieve the target-hitting tasks. By not guiding in the *z*-direction, we also reduced the potential masking effects of the guidance on the perception of the haptic rendering of the pendulum dynamics. The resulting equation for the PD controller is as follows:

4 $$\begin{aligned} F_{HG} = K_p e(t) + K_d\frac{d}{dt}e(t), \end{aligned}$$

where the *y*-axis error between the actual and the reference trajectory was denoted as *e*(*t*), and the proportional ($K_p$) and derivative ($K_d$) gains were set to 75.0 N/m and 15 N$\cdot$s/m, respectively. The guiding force was added to the haptic rendering force ($F_{rod}$ in Eq. (3)). The order of magnitude of the guidance force was around four times the haptic rendering.

### Participants

Forty-two unimpaired participants performed the experiment. Data from two participants were excluded from further analysis. One participant exhibited errors three standard deviations higher than the average of all participants. We encountered a technical problem when recording the data of a second participant, leading to missing data within the dataset. Thus, 40 participants were included in the analysis (age = $27 \pm 6\,yrs$; 19 identified as female, 21 identified as male; no participants identified as non-binary). The target sample size of approximately 40 participants was determined based on a power analysis, as detailed in Appendix A. Handedness was assessed using the Short-Form Edinburgh Handedness Inventory [46], resulting in 35 right-handed, four left-handed, and one ambidextrous participant. All participants signed the informed consent to participate in the study, which was approved by the TU Delft Human Research Ethics Committee (HREC).

Participants were allocated into two training groups: *Control* or *Experimental*. The *Experimental* group received haptic guidance during some parts of the training phase (see the Study protocol Section), while the *Control* group practiced without any physical assistance. To promote an even distribution between groups, we used an adaptive randomization method. We randomly allocated the first twenty participants into one of the two training groups and distributed new ones into each group based on their sex and results from the Locus of Control questionnaire (see the Outcome metrics Section), similar to [7]. The Locus of Control was employed as it directly relates to the perception of control, which aligned with the groups’ training conditions (guidance vs. no guidance).

### Study protocol

**Fig. 3:**
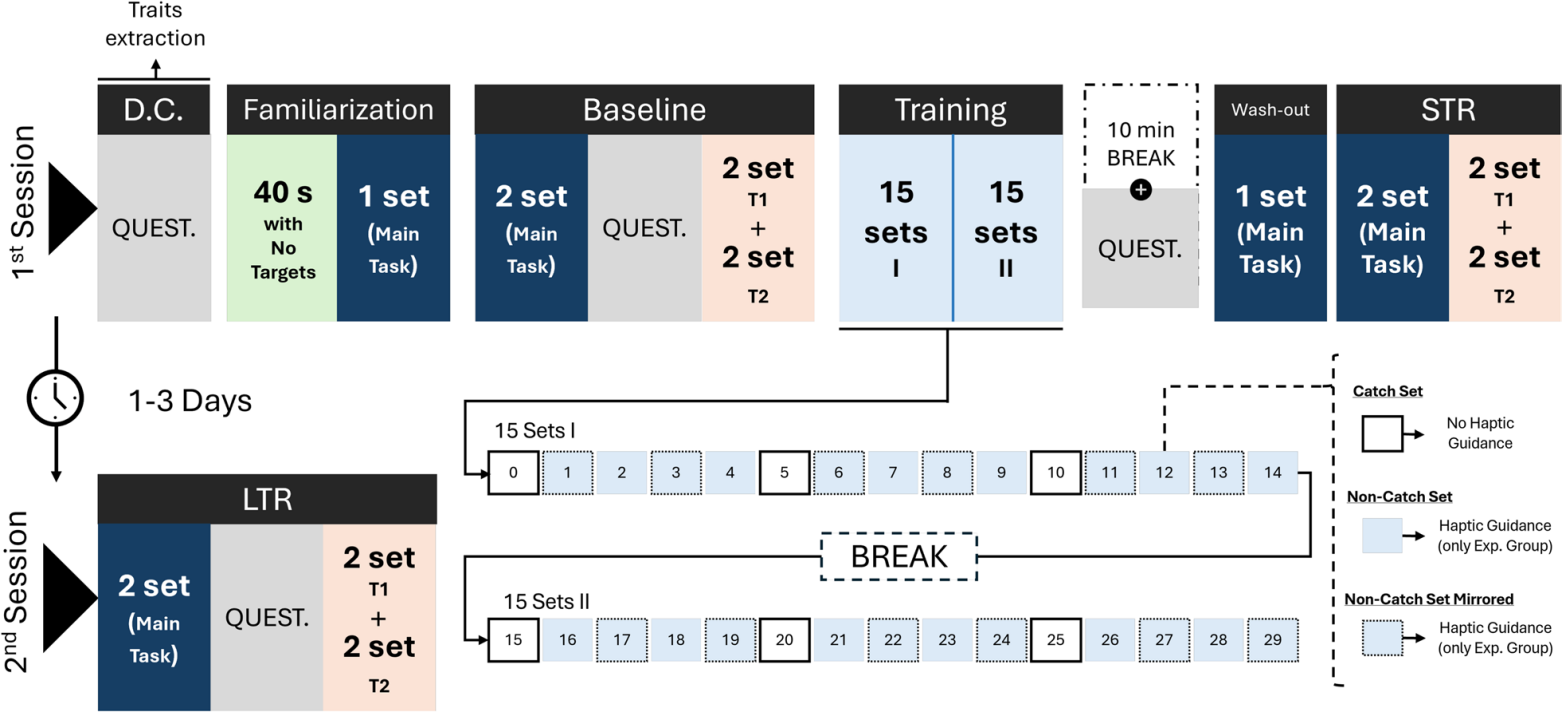
The study protocol included two sessions spaced by 1 to 3 days. A set comprised 20 targets. D.C.: Data collection, QUEST.: Questionnaires, s: Seconds, T1: Position transfer task, T2: Dynamics transfer task, STR: Short-Term retention, LTR: Long-Term retention, Exp.: Experimental

The experiment was conducted in two locations: Delft University of Technology, Delft, the Netherlands, and Alten Netherlands B.V., Rotterdam, the Netherlands. The experimental setup and protocol were identical in both locations. Minor environmental differences (e.g., room layout, lighting) were not expected to systematically affect performance. The overview of the experimental protocol can be found in Fig. 3.

The experiment took place in two sessions on different days, with one to three days between sessions, following recommendations to evaluate motor learning [3]. At the beginning of the first session, participants were invited to sit at the set-up table. The chair height was adjusted based on personal preferences to ensure a comfortable arm movement within the robot’s workspace. The haptic device was placed at a reachable distance with a relaxed posture on the dominant hand’s side. The screen was placed on the opposite side of the device in front of the participant. Participants were informed about the goal of the pendulum task at the beginning of the first session. No extra information was given during the rest of the experiment.

The experiment began by inviting participants to fill out the first block of questionnaires, including demographic data collection and the questionnaires to quantify the personality traits (see the Outcome metrics Section). Participants were then invited to familiarize themselves with the haptic device and the virtual environment for 40 s. During this familiarization phase, they were asked to move the pendulum freely in the virtual environment without loading any target. They could observe the pendulum moving and feel the haptic rendering of the pendulum dynamics through the haptic device end-effector. Once the 40 s were over, participants were instructed again about the game goal: move the pendulum such that the red ball hits each wall as close as possible to the target’s center. They were then invited to play a first set of 20 targets.

Once the familiarization was completed, the main experiment began. Participants underwent three main phases during Session 1: baseline, training, and short-term retention. During these phases, participants performed one or three different tasks. The *main task* consisted of playing 20 targets in a specific order. Each time the main task was played, targets were set in the same sequence of positions, except during the training phase, in which some of the sets were mirrored (further explained later in this section). Participants also played two transfer tasks to asses the generalization of the acquired skill. Those tasks were similar to the *main task* but included slight design variations. In the *position transfer task*, the targets were randomly re-located (still appearing every 1 s) to introduce new movement sequences. During the *dynamics transfer task*, the target positions were kept the same as during the *main task*, but the pendulum dynamics were changed. While the appearance of the pendulum did not change, the pendulum rod length was reduced by 70% in Eq. (1) and (3). This variation affected the pendulum’s natural frequency, which increased from 0.573 Hz to 0.685 Hz. For both transfer tasks, the goal remained the same: Use the pendulum mass to hit each target.

The baseline included two sets of 20 targets for the *main task*. The game did not stop between sets within the same task, but there was an extended pause of three seconds in which no new targets were loaded. After the second set was finalized, participants completed a new set of questionnaires to assess motivation and agency. To keep our analysis focused on answering the listed hypotheses, the analysis of motivation and agency are out of the scope of this work. Participants were then asked to play the game two more times, for two sets of the *position transfer task* and two sets of the *dynamics transfer task*, with an on-demand break offered between the two tasks.

After the baseline trials were complete, participants began the training. They completed two rounds of 15 sets of the main task of 20 targets each. Participants had the opportunity to take a break between rounds. During training, the *Experimental* group received haptic guidance on top of the haptic rendering. However, to avoid participant reliance on the guidance, guidance was removed during the first set and once every five sets (“catch sets”). In addition, to avoid only learning the specific movement patterns and target positions, mirrored sets were interspersed within the non-catch sets for both groups. The position of the targets during these sets was mirrored with respect to the walls’ *y*-axis. The distribution of “catch sets” and mirrored sets can be found in Fig. 3. Participants were informed that they might or might not be assisted during training to promote active participation. The *Control* group only experienced the haptic rendering from the pendulum dynamics during training.

Immediately following the last training set, participants took a 10-minute break. During this time, they filled in a new set of questionnaires. The *Experimental* group was asked questions about their subjective experience with the robotic guidance, i.e., how disturbing, frustrating or restrictive it was perceived (see the Outcome metrics Section). Following the break, a washout set of the main task was conducted by both groups to mitigate any temporary effects from training with haptic guidance, e.g., “slacking” [47].

Right after the washout set, participants performed the short-term retention phase. The structure was similar to the baseline but without the questionnaire. Participants returned after one to three days to perform a long-term retention phase, which was structured identically to the baseline tests.

### Outcome metrics

#### Personality traits questionnaires

Before the familiarization phase, participants completed a battery of questionnaires assessing the selected personality traits to study. These personality traits included the LOC scale [34], the Transform of Challenge and Transform of Boredom sub-scales from the Autotelic personality questionnaire [33], and the Achiever and Free Spirit sections of the Hexad Gaming style questionnaire [39]. All the questionnaires, except for the LOC, were formed by seven-point-based questions and normalized between 0 and 1 (low to high level of trait/characteristic). The LOC questionnaire was formed by 23 multiple-choice questions, and the overall score for the whole questionnaire ranged from 0 to 23. To improve interpretability and facilitate later modeling (see the Statistical analysis Section), this range was normalized from -1 to 1 to reflect the continuum between *Internal* LOC (-1) and *External* LOC (1), which are widely recognized classifications in literature and commonly used to interpret behavior. In addition, the LOC scores usually follow an approximate Gaussian distribution centered near zero. This makes this range statistically practical and close to the centered scale. Internal and external LOC differ in whether outcomes from an action are attributed to oneself or external circumstances, respectively. The employed questions for all the questionnaires can be found in the Appendix C.

#### Human-robot interaction experience questionnaire

Three questions were filled in by only the *Experimental* group after training. These questions related to frustration, disturbance, and restrictiveness perception during the training (see Appendix C). They were answered on a seven-point scale, which was then normalized between 0 and 1 (low to high).

#### Task performance: absolute error

To assess motor learning, the distance between the pendulum’s mass position and each target’s centerline at the time of pendulum-wall contact was calculated (|*Error*|), in meters. This was used as our performance metric and one value per wall was obtained.

#### Human-robot interaction: interaction force

To assess participants’ interaction with the haptic device, the human-robot interaction force was estimated. This estimate was computed using Reaction Torque Observers based on recorded motor currents and the robot dynamic model, as implemented in [44]. For the analysis, we used the average force per target in Newtons. We calculated this average force within the interval from consecutive midpoints between walls.

### Statistical analysis

To evaluate the hypotheses outlined in the Introduction Section, we used Linear Mixed Models (LMMs). These models were fitted using the $\texttt{lmer}$ function from the $\texttt{lmerTest}$ package in $\texttt{R}$. Statistical significance was set at $p < 0.05$, and *p*-values were adjusted for multiple comparisons using Bonferroni correction.

The employed LMMs were selected as outlined in Appendix D. We group them throughout the current section depending on the hypotheses they are tailored to evaluate. Table 1 summarizes the variables that can be included in the models. Task performance (|*Error*|) and human-robot interaction force (|*IntForce*|) metrics were analyzed as dependent variables depending on the model. Logarithmic transformations were applied to correct skewed distributions and achieve normality requirements.

**Table 1:** Variables employed for the LMM. Data was structured at the target level; However, in models where this structure was not applicable, the dataset was reduced to eliminate duplicate entries. The variables |Error| and |IntForce| were log-transformed to address skewness in their distributions

Variable	Type	Description	Values
*ID*	Int	Participant number	1 to 40
*wIndex*	Int	Wall number within a set	0 to 19
*Task*	Cat	$\textrm{Task type}^1$ (main task or transfer task)	mn, t1, t2
*Stage*	Cat	$\textrm{Experimental stage}^1$ (baseline, training, short- and long-term retention)	BL, TR, STR, LTR
*sIndex*	Int	Set identifier within a Task in a specific Stage	0 to ($N_{set}$-1)
$XX_c$	Cont	Normalized and mean-centered value of the trait score. XX are substituted by the corresponding personality trait.	0-$\overline{XX_{norm}}$ to 1-$\overline{XX_{norm}}$
|*Error*|	Cont	Absolute error values	–
|*IntForce*|	Cont	Interaction force values	–
*NewWalls*	Int	Wall number within the position transfer task	0 to 40

Int: Integer, Cat: Categorical, Cont: Continuous

^1^Depending on the model, some variables may not include all levels, e.g., when the training phase was excluded from a model, the variable “Stage” would only include BL, STR, and LTR

#### Models to infer motor learning (M1.1 and M1.2)

To evaluate the impact of personality traits and the training condition on motor learning outcomes across different experimental phases (related to hypotheses H1 and H2) we employed two models, one for each dependent variable. These models include independent variables regarding the training group, the task type, the stage, and the personality traits (see description in Table 1). Given the extensive number of variables and potential interactions, a stepwise comparison between models of different complexity was performed using the Akaike Information Criterion (AIC) and Bayesian Information Criterion (BIC) to prevent overfitting and ensure stability (see Appendix D).

For the performance metric (|*Error*|), the following model (M1.1) with the smallest AIC and BIC was chosen:

$$\begin{aligned} log_{{10}} |{\text{Error}}| & = Group \times (Task + Stage) \\ & + (TC_{c} + LOC_{c} ) \\ & \times (Task + Stage + sIndex) \\ & + Task \times Stage + Group \\ & \times TC_{c} \times Stage + (1|ID) \\ & + (1|wIndex:NewWalls). \\ \end{aligned} $$

In this equation, the normalized and centered results from the Transform of Challenge ($TC_c$) and Locus of Control (*LOC*_*c*_) were included as traits of interest, as others did not show statistical significance during model selection. Therefore, the hypothesis related to the Achiever gaming style (H1.3) is not supported by the data. A nested random effect for wall index (*wIndex* : *NewWalls*) was adjusted to account for changes in wall positioning during the position-based transfer task.

A similar model (M1.2) was developed for the interaction force metric. Notably, this model included an additional interaction term, $Group \times LOC_c \times Stage$. When compared with other configurations, the model with this extra interaction led to a lower AIC and slightly increased BIC (see Appendix D). In view of these competing results, previous literature was used to guide the choice. This extra relationship was considered of interest as LOC was found to correlate to the interaction force metric during the training phase when the haptic guidance was active (see [43]). The final model has the form:

$$ \begin{aligned} \log _{{10}} |{\text{IntForce}}| & = Group \times (Task + Stage) \\ & + (TC_{c} + LOC_{c} ) \\ & \times (Task + Stage + sIndex) \\ & + Task \times Stage + Group \times TC_{c} \\ & \times Stage + Group \times LOC_{c} \\ & \times Stage + (1|ID) \\ & + (1|wIndex:NewWalls). \\ \end{aligned} $$

Note that while this model does not include the Free Spirit gaming style, results from an alternative model (see Appendixes D and E) suggest a potential relationship between this trait and interaction force outcomes, which can be considered of interest for hypothesis H1.4. Yet, fully evaluating this effect would require studying the model complexity beyond the current study’s scope, complicating the understanding of the results. As such, we leave a thorough investigation of H1.4 for future work.    

#### Human-robot interaction perception model (M2)

To investigate whether subjective perceptions of human-robot interaction (HRI) influenced task performance (H3), we employed the following model:

$$ \begin{aligned} \log _{{10}} |{\text{Error}}| & = (AC_{c} + FS_{c} + TC_{c} + LOC_{c} ) \\ & \times HRIQuestion \times Stage \\ & + (1|ID) + (1|wIndex), \\ \end{aligned} $$

where the *HRIQuestion* represents the normalized and centered response to each of the specific HRI perception questions. The normalized and centered version of four of the traits were considered of interest for this model, i.e., the Achiever ($AC_c$) and Free Spirit ($FS_c$) gaming styles, Transform of Challenge ($TC_c$), and Locus of Control ($LOC_c$). All the phases were included in this dataset (baseline, training, short- and long-term retention) while the transfer tasks were excluded as HRI questions were asked exclusively after the training phase, which did not include the transfer tasks.

## Results

**Fig. 4:**
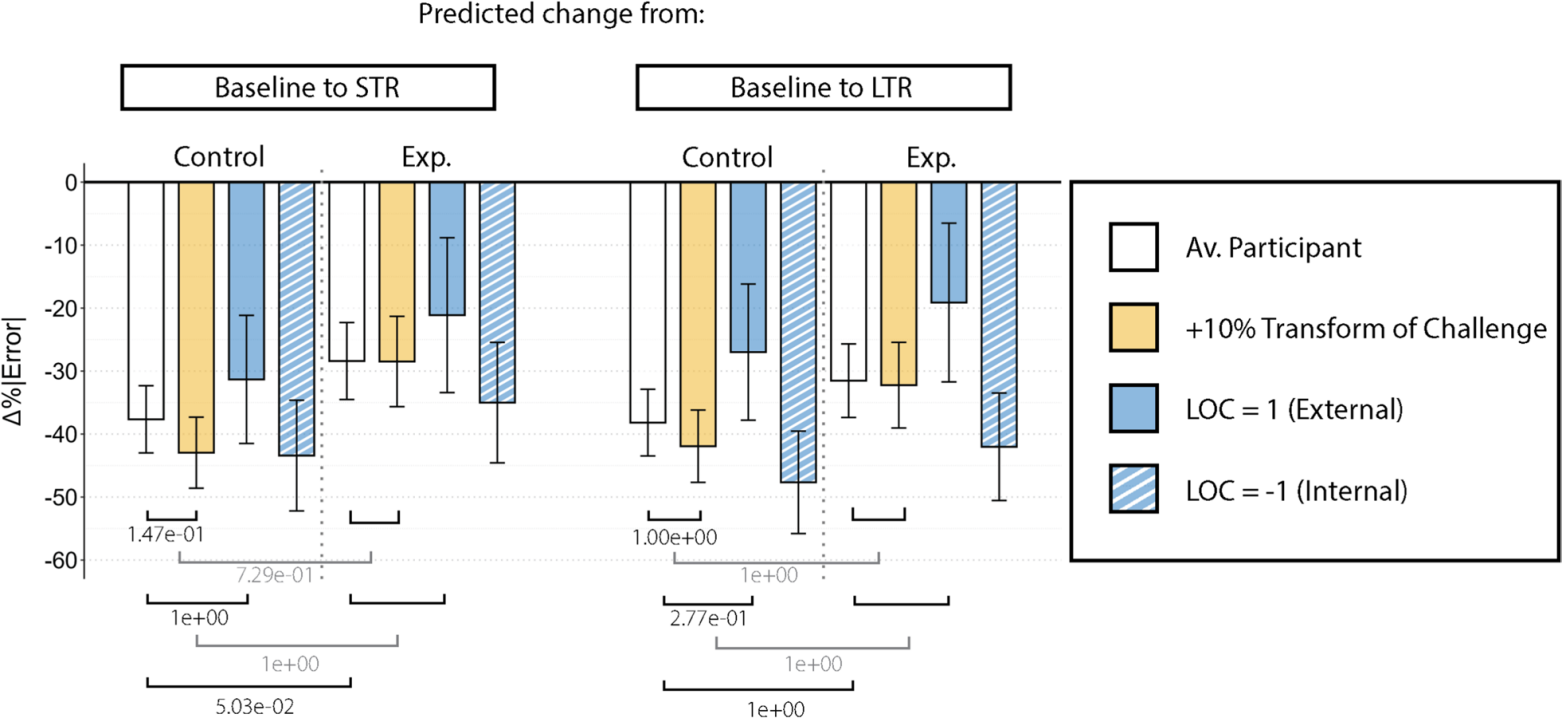
Barplot showing results from the M1.1 LMM regarding exclusively the main task. Each bar shows the error reduction that the model predicts a participant will show in different situations, i.e., from baseline to short-term retention (left) and from baseline to long-term retention (right). Note that the terms related to the influence of LOC in the *Experimental* group from baseline to STR or LTR were excluded from the model M1.1 to improve interpretability by reducing unnecessary complexity. White bars refer to a participant with average levels of every trait. Colored bars represent the same situation for a participant with a higher (solid) or lower (striped) level of a specific trait. Error bars represent the confidence interval associated with each estimated error reduction. The square brackets at the bottom represent the corrected *p*-values associated with those comparisons. Note that the ones in gray represent triple interactions; therefore, comparison of comparisons. STR: Short-term retention, LTR: Long-term retention, Av.: Average, Exp.: Experimental, LOC: Locus of Control

**Fig. 5:**
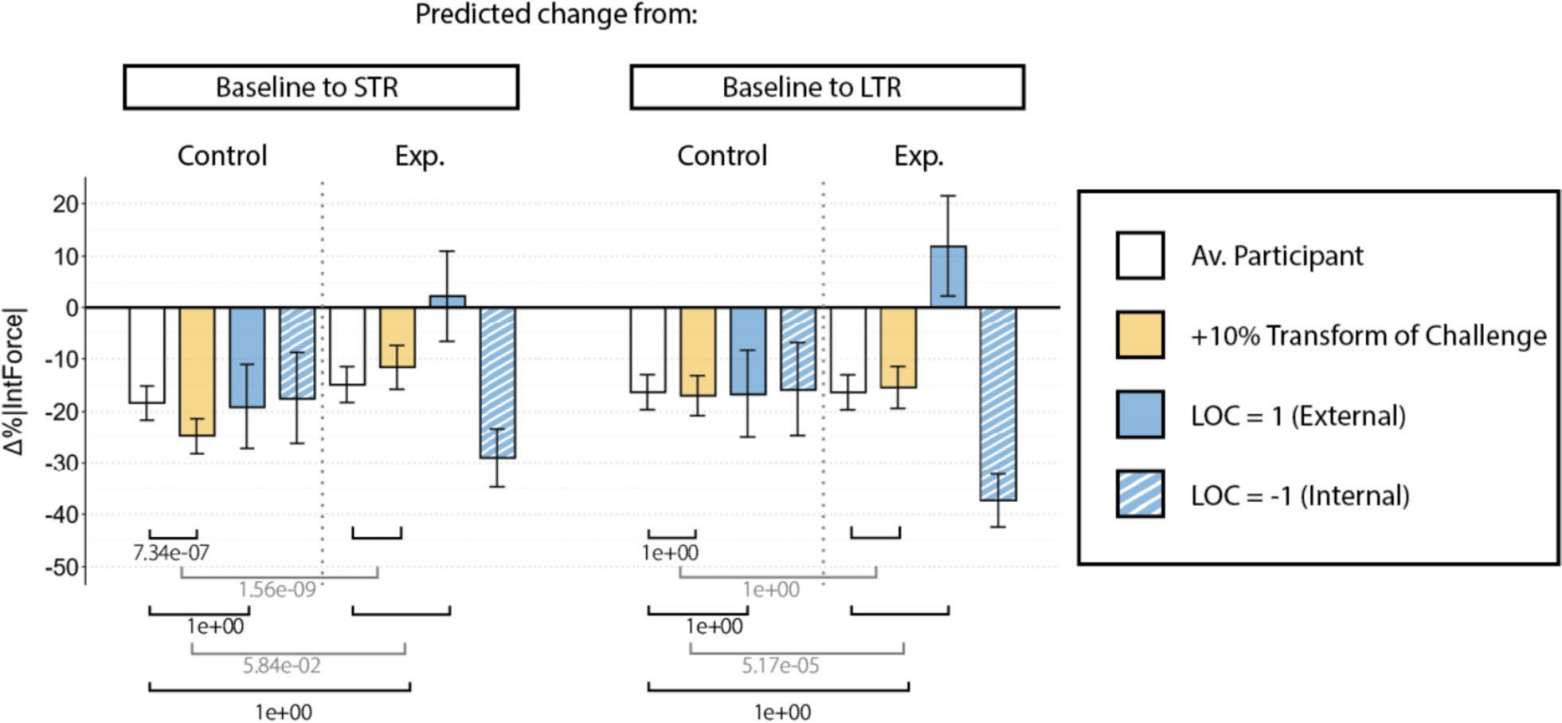
Barplot showing results from the M1.2 LMM regarding exclusively the main task. Each bar shows the interaction force reduction that the model predicts a participant will show in different situations, i.e., from baseline to short-term retention (left) and from baseline to long-term retention (right). White bars refer to a participant with average levels of every trait. Colored bars represent the same situation for a participant with a higher (solid) or lower (striped) level of a specific trait. Error bars represent the confidence interval associated with each estimated interaction force reduction. The square brackets at the bottom represent the corrected *p*-values associated with those comparisons. Note that the ones in gray represent triple interactions; therefore, comparison of comparisons. STR: Short-term retention, LTR: Long-term retention, Av.: Average, Exp.: Experimental, LOC: Locus of Control

**Fig. 6:**
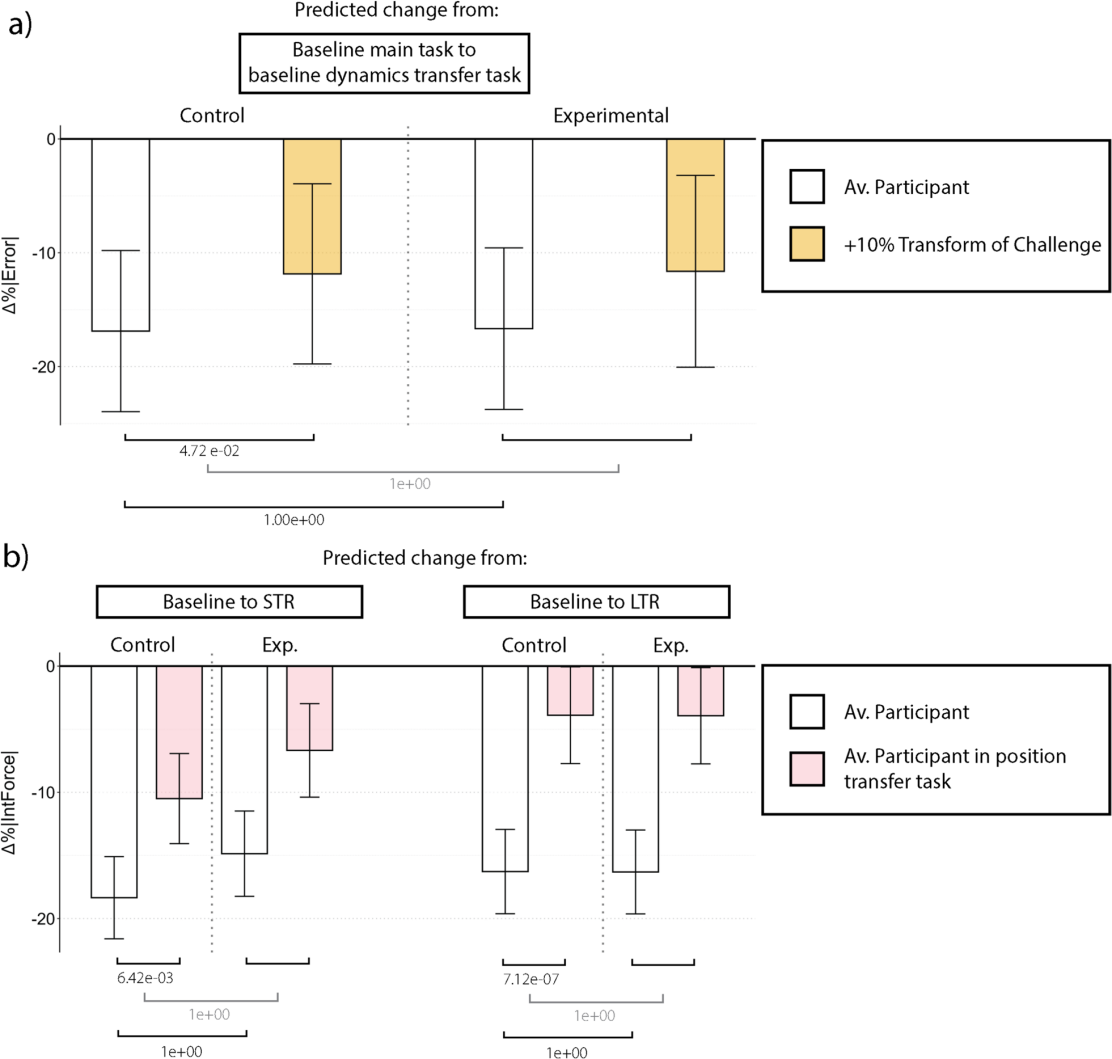
Top: Barplot showing results from the M1.1 LMM regarding the dynamics transfer task. Bottom: Barplot showing results from the M1.2 LMM regarding the position transfer task. Each bar shows the error (top) or interaction force (bottom) reduction that the model predicts a participant will show in different situations, i.e., from the main task in the baseline to the dynamics transfer task in the baseline (top) or from the baseline to STR/LTR in both, the main and the transfer task (bottom). White and pink bars refer to a participant with average levels of every trait. Other colored bars represent the same situation for a participant with a higher or lower level of a specific trait. Error bars represent the confidence interval associated with each estimated error or interaction force reduction. The square brackets at the bottom represent the corrected *p*-values associated with those comparisons. Note that the ones in gray represent triple interactions; therefore, comparison of comparisons. STR: Short-term retention, LTR: Long-term retention, Av.: Average

**Fig. 7:**
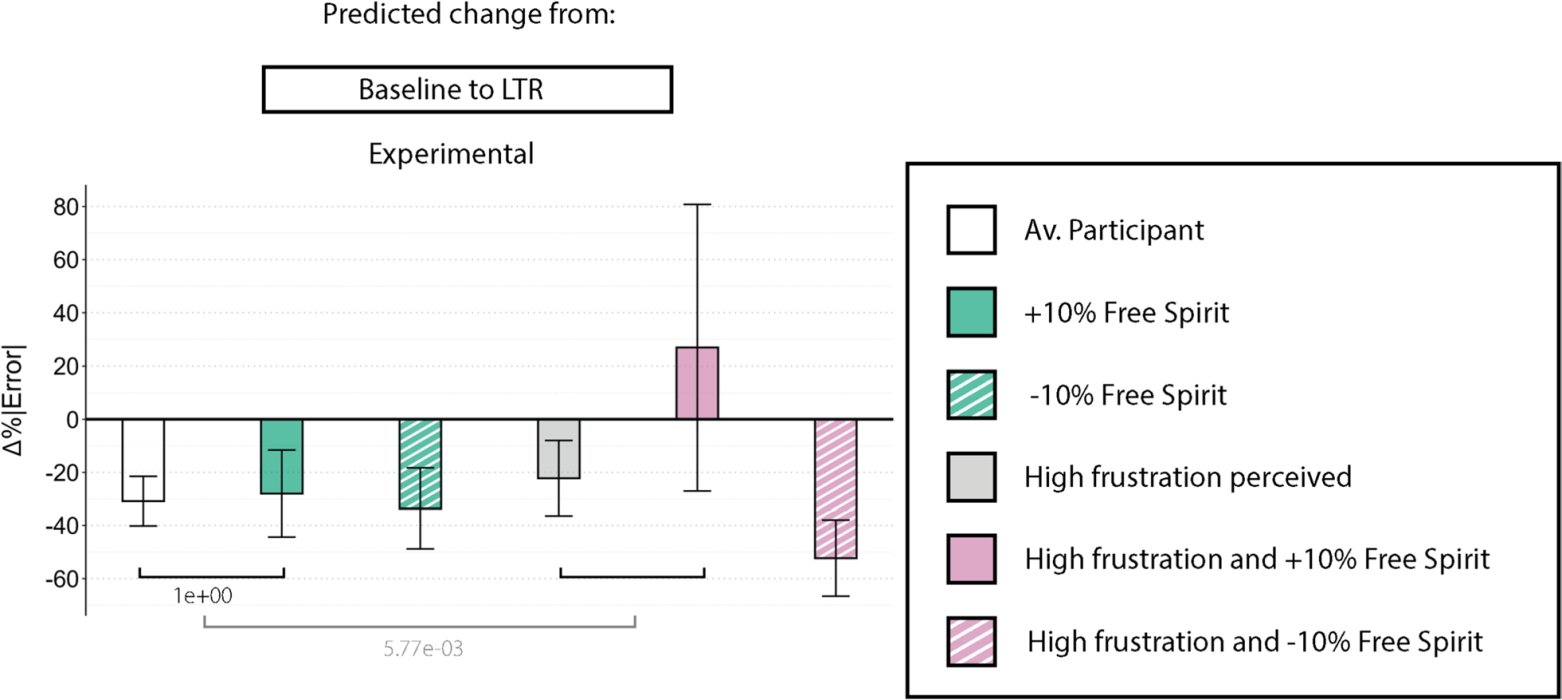
Barplot showing results from the M2 LMM, including only the *Experimental* group and the main task. Each bar shows the error reduction that the model predicts a participant will show in different situations, i.e., from baseline to long-term retention. The white bar refers to a participant with average levels of every trait. Colored bars represent the same situation for participants with a higher (solid) or lower (striped) level of a specific trait and/or high frustration. Error bars represent the confidence interval associated with each estimated error reduction. The square brackets at the bottom represent the corrected *p*-values associated with those comparisons. Note that the ones in white represent triple interactions; therefore, comparison of comparisons. LTR: Long-term retention, Av.: Average, Exp.: Experimental

The results from the LMM are visually summarized in Figs. 4, 5, 6 and 7, and tables with the full outputs from the models are available in the Appendix E. The figures within this section show bar plots illustrating expected changes in performance or interaction force between two conditions. These changes compare the relative difference between a reference case, e.g., a participant in baseline with average trait levels (or a specific trait value), and a case of interest, e.g., the same participant in a later phase. The expected performance or interaction force in these individual conditions was calculated based on the corresponding LMM estimates, with the differences reflecting the predicted percentage of improvement or decline. Furthermore, we provide 95% confidence intervals around these estimated changes to show the uncertainty around our estimates.

Note that some of the changes shown in the figures may not be significantly different from each other after applying the Bonferroni correction (see the corresponding corrected and uncorrected *p*-values in Appendix E). We decided to include them for consistent comparisons across conditions and to highlight emerging patterns that could guide future work.

Throughout this section, to illustrate the comparison cases, we refer to high or low levels of personality traits or gaming styles. We set those values to be 10% above (high level of a trait/gaming style) or below (low level of a trait/gaming style) the average participant levels. This percentage was selected as it represents a small, realistic shift within the observed data range, allowing us to meaningfully illustrate potential individual differences. This does not apply to the Locus of Control, in which we will compare external LOC (LOC = 1) or internal LOC (LOC = -1), as these well-established endpoints represent psychologically meaningful behavioral profiles. For all the cases, this is explicitly indicated in each figure.

### Motor learning results (M1.1 and M1.2)

Outputs from model M1.1 were used to calculate expected |*Error*| evolution across phases. Those predictions are shown in Fig. 4 (main task) and Fig. 6 *top* (transfer tasks). Outputs from the model M1.2 were used to calculate the expected |*InteractionForce*| evolution and are shown in Fig. 5 (main task) and Fig. 6 *bottom* (transfer tasks).

When focusing on the *Control* group, Fig. 4 illustrates how a participant with average levels of every personality trait (av. participant) is expected to show an error reduction of 38% from baseline to short-term retention (STR) and 39% from baseline to long-term retention (LTR) if completing the main task.

Regarding the effect of personality traits in the *Control* group, a higher Transform of Challenge reduces even more the error from baseline to STR/LTR compared to an average participant; i.e., these participants showed improved performance. However, the *p*-values associated with these effects were only significant before Bonferroni’s correction (after the correction: $p = 0.147$ and $p=1$ respectively). A high level of this trait corresponded with around 5% performance improvement in addition to the error reduction expected from an average participant from baseline to STR. LOC also slightly influenced performance changes from baseline to LTR in the *Control* group (*p*-value only significant before Bonferroni’s correction, after: $p=0.277$). An external LOC was associated with degraded performance from baseline to LTR compared to an average participant from baseline to LTR.

The *Experimental* group exhibits a degraded performance metric, with reductions of around 28% from baseline to STR (corrected *p*-value: $p=0.05$) and around 31.5% from baseline to LTR (corrected *p*-value: $p=1$). Those values can be compared to the 38% (STR) and 39% (LTR) error reduction seen in the *Control* group. In the *Experimental* group, a high level of the Transform of Challenge trait did not show better improvement compared with the improvement shown in the *Control* group (Corrected *p*-values for the STR and LTR were $p=0.729$ and $p=1$ respectively).

We found that the human-robot interaction force was correlated with personality traits. Figure 5 shows how high levels of Transform of Challenge were associated with different changes from baseline to STR depending on the training group. Participants in the *Control* group with higher levels of this trait exhibited a larger reduction in the interaction forces from baseline to STR, compared to participants with this trait in the *Experimental* group (corrected *p*-value $p=1.56e^{-9}$). For participants allocated to the *Experimental* group, LOC also had a notable influence. While participants with an internal LOC showed larger interaction force reductions from baseline to STR and LTR, those participants with external LOC demonstrated a force increase. In this case, the *p*-values associated with both personality trait effects showed statistically significant after the Bonferroni correction ($p=5.84e^{-2}$ and $p=5.17e^{-5}$ respectively).

Results regarding the transfer tasks can be found in Fig. 6. Both metrics (performance and interaction force) were affected by the transfer task. Some tendencies were found in the *Control* group. Participants in this group showed better performance in the dynamics transfer task w.r.t. the main task during baseline ($p=6.303e^{-4}$). Participants with high levels of Transform of Challenge in the same group showed smaller differences ($p=4.719e^{-2}$). The position-based transfer task was associated with differences in the interaction force metric. In particular, participants in the *Control* group reduced, to a smaller degree, the interaction force from baseline to STR and LTR in the position transfer task as compared to the main task ($p=6.417e^{-3}$ and $p=7.124e^{-7}$ respectively).

### Human-robot interaction perception results (M2)

We did not find significant effects between the subjective experience of the haptic guidance (HRI questions) and changes in performance or interaction force from baseline to any of the other phases. However, we found that a high frustration combined with a high Free Spirit will cause a significantly higher error in the long-term retention (LTR) for those participants allocated in the *Experimental* group ($p=5.772e^{-3}$), i.e., they showed degraded performance metric. While participants in this group showed error reduction from baseline to LTR, this improvement was no longer statistically significant when considering participants with both high frustration and high Free Spirit scores. Interestingly, those participants who perceived high frustration but scored lower in the Free Spirit gaming style questionnaire showed an error reduction of almost 20% more than an average participant when comparing baseline to LTR (Fig. 7).

## Discussion

### The influence of personality traits in motor learning (H1)

Overall, participants demonstrated learning progress, i.e., reduced the error and interaction forces from baseline to STR and LTR. We observed slightly lower learning for those participants who trained with haptic guidance (*Experimental* group) compared to the ones who trained without it (*Control* group). Still, the *p*-values associated with these effects did not remain statistically significant after Bonferroni correction, contrary to our first hypothesis (H1.1). This aligns with our previous findings in a similar pendulum task, where we did not find significant differences between training with haptic guidance and without it in terms of accuracy improvements after training [44]. Yet, although the power analysis (Appendix A) confirmed that 40 participants were enough to detect the effect size, it could be that we overestimate that effect. This effect might be too small to be detected for this sample size; repeating the experiment with a larger sample size is left for future work.

From the evaluated personality traits and gaming styles, only the Transform of Challenge (H1.2) and Locus of Control (H1.5) were found to influence motor learning. The Achiever gaming style was excluded from the LMM M1.1, as its inclusion did not improve model fit and resulted in higher AIC and BIC values. Therefore, hypothesis H1.3 is not supported by the data. Although exploratory analyses suggested a potential relationship relevant to H1.4 (related to the Free Spirit gaming style), fully evaluating this effect would require studying the model complexity beyond the current study’s scope. As such, we leave a thorough investigation of H1.4 for future work (see Appendices D and E for completeness).

#### The effect of autotelic personality in motor learning depends on the training strategy (H1.2)

In line with our second hypothesis (H1.2), we observed that those scoring high in Transform of Challenge showed different patterns in both performance and human-robot interaction force changes from baseline to STR depending on the training group they were allocated to. Those with high Transform of Challenge levels in the *Control* group exhibited slightly greater improvement in performance (lower error) from baseline to STR when compared to those with average trait levels. However, this effect did not remain statistically significant after applying Bonferroni correction to the specific cases, likely due to the conservative nature of the adjustment and the small sample size. However, differences remained significant after the Bonferroni correction when evaluating the interaction force. Participants with a high level of Transform of Challenge in the *Control* group reduced the interaction force substantially more from baseline to STR than those in the *Experimental* group. This greater force reduction in the *Control* group is in line with literature that shows an association between Autotelic behaviors and high-intensity athletes [32], who are experts in movement economy and efficiency [48]. For these subjects moved by challenges, the haptic guidance may have reduced their perception of the task difficulty, leading to a smaller reduction of the interaction force in those allocated in the *Experimental* group. The haptic guidance may have interfered with this group of participants’ engagement and focus, making the learning environment potentially less ideal for this personality type. This aligns with our observations during training: Participants with high Transform of Challenge demonstrated worse performance under guidance than average participants, likely due to a lack of engagement caused by the reduced perceived difficulty [43].

These findings suggest that individuals with Autotelic traits, particularly Transform of Challenge, could excel in economizing their effort. However, they may be sensitive to interventions that reduce the perceived challenge, such as haptic guidance. That finding positions this trait as an intriguing subject for further study. Future research should explore the interplay between this trait and perceived difficulty and attentional focus. Additionally, employing methods like error amplification, which increases task difficulty, could help sustain the motivation of such participants and further enhance their learning outcomes [7].

#### The effect of locus of control in motor learning depends on the training strategy (H1.5)

In line with our hypothesis H1.5, we found a significant relationship between the LOC and the reduction in human-robot interaction forces from baseline to the retention phases. For participants in the *Experimental* group, we found contrasting tendencies in interaction force reduction after training depending on whether participants exhibited a more external or internal LOC orientation. In particular, participants with an external LOC (i.e., believe that outcomes from actions are attributed to external circumstances) increased their interaction force between baseline and STR & LTR. In contrast, those with an internal LOC (i.e., believe that outcomes from actions are attributed to oneself) demonstrated significantly larger reductions when compared to participants with average trait levels allocated in the *Experimental* group.

These results align with our previous findings during training: Participants in the *Experimental* group with external LOC exhibited higher interaction forces during training than those with internal LOC [43]. A possible explanation is that participants with an internal LOC perceived the haptic guidance as a means to enhance their personal control over the pendulum dynamics, which aligns with their intrinsic belief in self-control. By attributing successful performance improvements to their own actions, internal LOC participants may have been more engaged in refining their interaction strategy. This interpretation is consistent with previous studies showing that individuals with an internal LOC tend to perform better when provided with positive feedback, or in this case, perceiving better performance by obtaining a higher score while receiving guidance [35].

Conversely, participants with a more external LOC may have perceived the haptic guidance as the primary driver of task success, leading to less motivation to actively improve their performance/interaction with the device. Prior research in educational and behavioral psychology has shown that individuals with an external LOC often demonstrate lower persistence when facing new challenges or scenarios [49]. In the context of haptic guidance, this may translate to a tendency to “lean on” the robotic support, resulting in higher interaction forces from baseline to retention phases. However, this was not accompanied by a statistically significant decline in performance. One interpretation is that these participants, having attributed earlier improvements to the robotic guidance, may have been unprepared for its removal, resulting in inefficient strategies that increased physical interaction with the device without necessarily worsening performance outcomes.

### The type of transfer task affects performance and interaction metrics (H2)

When examining the performance and interaction metrics during the transfer tasks, distinct patterns emerged. During baseline, participants exhibited smaller errors in the different dynamics transfer task as compared to the main task, in line with our hypothesis (H2.2). The dynamics transfer task involved a pendulum with a shorter rod, resulting in a higher natural frequency. Since the hand movement requirements remained unchanged (i.e., identical target positions as in the main task), the altered dynamics may have made it easier for participants to move outside the natural frequency of the pendulum. Prior work from our group showed that deviation from the pendulum’s natural frequency is indeed correlated with higher control in this particular target-hitting task [44]. The improved control could have contributed to better performance. Interestingly, those participants with a high Transform of Challenge score showed smaller error reduction from the main to the dynamics transfer task in baseline. This suggests that these individuals may have perceived the altered dynamics task as less difficult and, therefore, less engaging, in line with hypothesis H2.3 and the previously discussed findings related to motor learning. The better performance observed in the dynamics transfer task compared to the main task was no longer significant in the subsequent STR and LTR phases. This likely reflects participants’ increased mastery of the main task, which was the only task trained during the training phase. As a result, improved overall skill during the training phase may have reduced the performance gap between the two tasks.

Contrary to hypothesis H2.1, we did not find differences in performance or interaction force between the main and the position-based transfer task during baseline. However, the human-robot interaction force was reduced less in the position-based transfer task than in the main task when comparing the baseline to STR in both cases. This transfer task was designed to promote wider movements, obligating the trainees to deal with bigger pendulum amplitudes. This could have hindered the generalization process, as they needed to increase the interaction force to overcome the challenge.

### The interaction between personality traits and human-robot interaction perception affect performance (H3)

Participants in the *Experimental* group were asked three additional questions about how restricting or permitting they found the guidance, how disturbing or helpful they found the guidance, and their level of frustration during the task (HRI questions). Contrary to our hypothesis H3.1, none of the questions showed a statistically significant effect on performance improvement. Yet, when the information about personality traits was included in the analysis, we found that participants with varying levels of the Free Spirit gaming style were particularly sensitive to frustration, affecting their performance and aligning with hypothesis H3.2.

The performance improvement from baseline to long-term retention in the *Experimental* group — potentially associated with learning effects — was no longer statistically significant when accounting for participants with both high frustration and high Free Spirit scores. Individuals with high Free Spirit gaming style scores — characterized by strong exploratory tendencies [41] — did not experience a performance improvement from baseline to long-term retention if they felt frustrated. This might be due to the fact that the guidance restricted their exploration tendencies. The Free Spirit gaming style has been found to negatively correlate with social design game elements, suggesting a preference for autonomy and self-expression over structured interactions [50]. In contrast, participants who reported high frustration but scored low in Free Spirit showed greater performance improvements from baseline to LTR. With a lower tendency toward exploration, these participants with a low Free Spirit gaming style may have been less inclined to deviate from the guidance and more likely to adhere to the training structure. Consequently, their frustration may have driven them to focus on refining familiar, learned movements rather than seeking alternative approaches, ultimately enhancing their task performance over time. These findings also align with prior research in human-in-the-loop systems, which emphasizes the importance of incorporating human preferences to maintain optimal human performance [20, 21].

### Implications for robot-aided motor learning and rehabilitation and future work

Our findings suggest that autotelic personality, Locus of Control, and the Free Spirit gaming style may shape how individuals respond to haptic guidance, highlighting the need for personalized robot-aided rehabilitation protocols. Understanding and extending our knowledge about how these trait-dependent differences interact with robotic assistance could help refine robotic rehabilitation approaches by tailoring assistance levels and feedback mechanisms to enhance performance and motor (re)learning. Personality traits and individuals’ desires and needs might change before and after an acquired brain injury and through the recovery process. Yet, we expect these changes to follow a slowed temporal scale compared to changes in mental states, which vary largely depending on the day, the time of the day, or the task to be completed, reducing the need for *in-vivo* measurements to provide tailored rehabilitation programs.

Although this work represents a first step toward understanding the potential value of incorporating personality traits into personalized rehabilitation protocols, future work should examine whether similar personality-dependent effects influence motor recovery in individuals post-stroke. This entails broadening the evaluated traits, as stroke frequently induces new or altered psychological traits that can be of importance when regulating their interaction with robotic devices [51]. Additionally, future studies could explore the relationship between personality traits and psychological states like perceived difficulty, attention focus, and engagement across diverse tasks and haptic training methods such as error-enhancing strategies, in both unimpaired and clinical populations.

### Study limitations

This study has several limitations. Although the statistical power computation showed that 40 participants were sufficient to detect some effects of interest, the model selection process led to the inclusion of more complex interaction terms than originally anticipated. As a result, the effective power of the statistical tests was reduced, which is reflected in the confidence intervals and non-statistically significant *p*-values observed in some results. This suggests that, while 40 participants is a reasonable sample size within motor learning experiments, it may be relatively small when investigating more complex effects, such as those involving personality traits and their interactions. Future studies could address this limitation by using the findings presented here to inform the design of future experiments, with larger sample sizes or simplified models to maintain adequate power.

Additionally, the values used to estimate high or low levels of personality trait effects in this work may not fully represent real-world distributions. Moreover, participants were not classified into distinct personality groups but rather exhibited a mix of traits simultaneously, making it difficult to isolate individual effects. Finally, we acknowledge that individual differences in motor learning may be shaped by a range of sociocultural influences, including those related to gender identity. We did not investigate gender-related effects in this study due to the already high complexity of the models. Yet, we encourage future work to explore this further using more inclusive and representative gender frameworks.

## Conclusion

This study highlights the multifaceted relationship between personality traits, motor learning, and human-robot interaction (HRI) in the context of robotic-assisted training. We conducted a motor learning experiment with forty unimpaired participants, half of whom received physical guidance from a robotic device. We found that trainees with high Transform of Challenge personalities showed less error reduction after training with haptic guidance, probably due to a lower perceived task difficulty. In addition, those with high Transform of Challenge characteristics who trained without haptic guidance improved their interaction with the robot as compared to the average participants. We theorize that participants with Autotelic personalities hold a high capacity to reduce effort but are substantially penalized by a loss of interest due to tasks that are too easy. Locus of Control (LOC) also showed the nuanced impact of personality on human-robot interaction. Internal LOC participants exhibited a substantial reduction in the human-robot interaction force after training. In contrast, external LOC participants demonstrated only minimal improvement, underscoring the interplay between perceived control and the effectiveness of guidance systems. Finally, participants with a low Free Spirit gaming style showed sensitivity to the subjective perception of frustration due to overly restrictive haptic guidance, resulting in an improvement in their performance after training.

Our work contributes to the understanding of how personality traits and human-robot interaction perception affect motor learning to inform the design of future personalized rehabilitation systems.

## Data Availability

The dataset employed in this study can be found online in the following repository: 10.5281/zenodo.16539273.

## Appendix A Power analysis

To ensure the study was adequately powered to detect the expected effects, a preliminary power analysis was conducted. We began by reviewing the relevant literature to estimate a suitable sample size. Özen et al. [44], who employed a similar experimental approach and set up, obtained statistically significant results with 40 participants. We adopted this number as an initial reference point for evaluating the statistical power in our study.

To verify the adequacy of this sample size, we generated several synthetic datasets simulating 40 participants. Expected effect sizes were informed by prior research in motor learning. However, effect sizes for personality traits had to be determined and chosen based on theoretical expectations and plausible assumptions, due to the absence of prior data linking personality traits with robotic training and motor learning. Remarkably, some of those estimated values were designed based on the hypotheses presented in the Introduction Section. A summary of all parameters used in the simulation, including expected performance improvements and standard deviations, is presented in Table 2.

**Table 2:** Values employed to create the synthetic data. Negative values mean a reduction in the Absolute error, therefore better performance

Parameter	Value	Source/Justification
Mean Absolute Error in baseline	0 m	Based on pilot
Pct Change STR Av. Participant	$-25\%$	As seen in [44]
Pct Change LTR Av. Participant	$-20\%$	As seen in [44]
Pct Change Exp Group Av. Participant	$+10\%$	As seen in [44]
Pct Extra Change AC (STR/LTR)	$-10\%$(C), $0\%$(E)	Hypothesis
Pct Extra Change FS (STR/LTR)	$+15\%$(C), $0\%$(E)	Hypothesis
Pct Extra Change TC (STR/LTR)	$+20\%$(C), $-20\%$(E)	Hypothesis
Pct Extra Change TB (STR/LTR)	$+10\%$(C), $0\%$(E)	Hypothesis
Pct Extra Change LOC (STR/LTR)	$+10\%$(C), $-10\%$(E)	Hypothesis
Mean Quest Score AC (0 to 1)	$0.7 \pm 0.15$	Based on prior experiments
Mean Quest Score FS (0 to 1)	$0.65 \pm 0.15$	Based on prior experiments
Mean Quest Score TC (0 to 1)	$0.65 \pm 0.15$	Based on prior experiments
Mean Quest Score TB (0 to 1)	$0.5 \pm 0.2$	Based on prior experiments
Mean Quest Score LOC (-1 to 1)	$0 \pm 0.5$	Based on prior experiments
Random Effect ID	$0 \pm \log _{10}(1.05)$	Hypothesis based on [44]
Random Effect wIndex	$0 \pm \log _{10}(1.02)$	Hypothesis
Residual Error SD	$\log _{10}(1.10)$	Hypothesis
Number of Participants	40	Sample size for simulation
Number of Simulations	200	Commonly used

Pct: Predicted, STR: Short-therm retention, LTR: Long-therm retention, C: Control, E: Experimental, AC: Achiever, FS: Free Spirit, TC: Transform of Challenge, TB: Transform of Boredom, LOC: Locus of Control, wIndex: Wall Index, ID: Identifier, SD: Standard deviation

Among the traits considered, we hypothesized that only the Transform of Challenge and Locus of Control would significantly interact with the group allocated. This expectation was based on theoretical reasoning: the guidance intervention was designed to enhance perceived control and, therefore, might also affect the challenge perception. Although one could argue that guidance may influence Free Spirit, we excluded this interaction under the rationale that it was not clear whether those participants would perceive the guidance as restrictive or as another element to explore. Based on these considerations, we specified the following linear mixed-effects model to simulate the outcome structure:

$$\begin{gathered} \log _{{10}} |{\text{Error}}| = {\mkern 1mu} (AC_{c} + FS_{c} + TC_{c} + TB_{c} + LOC) \hfill \\ \,\,\,\,\,\,\,\,\,\,\,\,\,\,\,\,\,\,\,\,\,\,\,\,\,\,\,\,\,\, \times Stage + (TC_{c} + LOC) \times Stage \hfill \\ \,\,\,\,\,\,\,\,\,\,\,\,\,\,\,\,\,\,\,\,\,\,\,\,\,\,\,\,\,\, \times Group{\mkern 1mu} + (1|ID) + (1|wIndex), \hfill \\ \end{gathered} $$

where each trait (e.g., $AC_{c}$ for Achiever) was centered before inclusion. *Stage* denotes the learning phase (baseline, short-term retention, or long-term retention), and *Group* indicates experimental condition (Control or Experimental). Random intercepts were included for participants (*ID*) and wall index within set (*wIndex*). The dependent variable was the base-10 logarithm of absolute error, computed as described in the Outcome metrics section. The decision to use a logarithmic scale was informed by prior experimental studies conducted within our lab, where error distributions were typically right-skewed. Given that similar motor learning tasks often yield skewed performance metrics, we adopted this transformation as a precautionary step grounded in empirical precedent.

The simulation code and datasets are publicly available via Zenodo, ensuring reproducibility. Power estimates (where the p-values have been adjusted using the Benjamini–Hochberg procedure) for each interaction of interest, along with their justification, are provided in Table 3.

**Table 3:** List of interaction effects of interest and associated statistical power

Interaction	Power	Justification/Reason of inclusion
Stage STR	$100\%$	Detecting Learning Effect
Stage LTR	$100\%$	Detecting Learning Effect
Stage STR:ExpGroup	$100\%$	Detecting Guidance Effects on Learning
Stage LTR:ExpGroup	$100\%$	Detecting Guidance Effects on Learning
$AC_{c}$:StageSTR	$70\%$	Detecting $AC_{c}$ Effect in STR in Control Group
$AC_{c}$:StageLTR	$67.5\%$	Detecting $AC_{c}$ Effect in LTR in Control Group
$FS_{c}$:StageSTR	$85.5\%$	Detecting $FS_{c}$ Effect in STR in Control Group
$FS_{c}$:StageLTR	$84\%$	Detecting $FS_{c}$ Effect in LTR in Control Group
$TC_{c}$:StageSTR	$95\%$	Detecting $TC_{c}$ in STR in Control Group
$TC_{c}$:ExpGroup:StageSTR	$94\%$	Detecting $TC_{c}$ Effect in STR in Experimental Group
$TC_{c}$:StageLTR	$93\%$	Detecting $TC_{c}$ Effect in LTR in Control Group
$TC_{c}$:ExpGroup:StageLTR	$94\%$	Detecting $TC_{c}$ Effect in LTR in Experimental Group
$TB_{c}$:StageSTR	$93.5\%$	Detecting $TB_{c}$ Effect in STR in Control Group
$TB_{c}$:StageLTR	$94\%$	Detecting $TB_{c}$ Effect in LTR in Control Group
LOC:StageSTR	$100\%$	Detecting LOC Effect in STR in Control Group
LOC:ExpGroup:StageSTR	$100\%$	Detecting LOC Effect in STR in Experimental Group
LOC:StageLTR	$100\%$	Detecting LOC Effect in LTR in Control Group
LOC:ExpGroup:StageLTR	$100\%$	Detecting LOC Effect in LTR in Experimental Group

Considered powerful for *p*-value<0.05 and adjusted using the Benjamini–Hochberg procedure. STR: Short-therm retention, LTR: Long-therm retention, ExpGroup: Experimental grouop, $AC_{c}$: Achiever centered, $FS_{c}$: Free Spirit centered, $TC_{c}$: Transform of Challenge centered, $TB_{c}$: Transform of Boredom centered, LOC: Locus of Control

## Appendix B Haptic guidance

The haptic guidance strategy used in this study was an adapted version of the approach introduced in  [44]. To realize the haptic guidance, we combined two elements. First, we employed the ACADO toolkit  [52] to compute the optimal reference trajectory for the pendulum’s end-effector. A proportional-derivative (PD) controller was then used to generate the forces required to minimize deviations between the device’s end-effector position and the reference trajectory along the *y*-axis (parameters explained in the main manuscript). The cost function used for optimization in the ACADO toolkit  [52] is:

$$\begin{aligned} & J(x,u) = \sum _ { k = t_0 } ^ { t_0 + N - 1 } h \left( x _ { k }, u _ { k } \right) ^ { \top } W h \left( x _ { k }, u _ { k } \right) \\ & \quad +h _ { t_0 + N } \left( x _ { t_0 + N } \right) ^ { \top } W _ { t_0 + N } h _ { t_0 + N } \left( x _ { t_0 + N } \right) , \end{aligned}$$

where $t_0$ is the initial time step, *h* and $h_{t+N}$ represent the stage cost function and terminal cost function, respectively. In these functions, the criteria to be minimized during optimization are defined. The initial time-step $t_0$ is selected to be exactly one second before the next wall is hit. This hit occurs at the terminal time step $t_0+N$. *N* is the horizon length and is set to 80 steps (12.5 ms per step). $u_k$ is the control action, $x_k$ is the system state at time step *k*, and $x_{t+N}$ is the terminal state. *W* and $W_{t+N}$ are cost matrices that assign weights to different terms in the cost function.

For this system, the stage cost function *h* and the terminal cost function $h_{t+N}$ are defined as follows:

$$h(x_{k} ,u_{k} ) = \left[ {\begin{array}{*{20}c} {y_{k} + l\sin (\theta _{k} )} \\ {z_{k} - l\cos (\theta _{k} )} \\ {\mathop {y_{k} }\limits^{.} + l\mathop {\theta _{k} }\limits^{.} \cos (\theta _{k} )} \\ {\mathop {z_{k} }\limits^{.} + l\mathop {\theta _{k} }\limits^{.} \sin (\theta _{k} )} \\ {F_{{HG_{y} }} } \\ {F_{{HG_{z} }} } \\ \end{array} } \right]$$

$$h_{{t_{0} + N}} (x_{{t_{0} + N}} ) = \left[ {\begin{array}{*{20}c} {y_{{t_{0} + N}} + l\sin (\theta _{{t_{0} + N}} ) - y_{{target}} } \\ {z_{{t_{0} + N}} - l\cos (\theta _{{t_{0} + N}} )} \\ {\dot{y}_{{t_{0} + N}} + l\dot{\theta }\cos (\theta _{{t_{0} + N}} )} \\ {\dot{z}_{{t_{0} + N}} + l\dot{\theta }\sin (\theta _{{t_{0} + N}} )} \\ \end{array} } \right]$$

In the cost function *h*, the first four rows describe the state of the ball (pendulum mass) at the time step *k*, expressed in terms of the robot end-effector position and velocity along the *y*- and *z*-axes. The last two rows represent the control actions, i.e., the forces applied to the end-effector by the robotic system at time step *k*, which influence the system dynamics. The terminal cost function $h_{t_0+N}$ represents the final state at the terminal time step. Notably, force terms are omitted here, as no further control action is required beyond this point in the optimization process. The first row includes $y_{target}$ to ensure that the system penalizes deviations from the target position along the *y*-axis.

The costs matrices *W* and $W_{t+N}$ are structured as follows:

$$ \begin{gathered} W = \left[ {\begin{array}{*{20}c} 0 & 0 & 0 & 0 & 0 & 0 \\ 0 & 0 & 0 & 0 & 0 & 0 \\ 0 & 0 & {Q_{{vy}} } & 0 & 0 & 0 \\ 0 & 0 & 0 & {Q_{{vz}} } & 0 & 0 \\ 0 & 0 & 0 & 0 & {R_{y} } & 0 \\ 0 & 0 & 0 & 0 & 0 & {R_{z} } \\ \end{array} } \right] \hfill \\ W_{{t_{0} + N}} = \left[ {\begin{array}{*{20}c} {Q_{{target}} } & 0 & 0 & 0 \\ 0 & 0 & 0 & 0 \\ 0 & 0 & {Q_{{vy}} } & 0 \\ 0 & 0 & 0 & {Q_{{vz}} } \\ \end{array} } \right]{\text{ }} \hfill \\ \end{gathered} $$

Each element in these matrices is designed to achieve specific optimization goals and chosen based on previous literature  [44, 53]:

- $Q_{vy}$ and $Q_{vz}$ penalize the velocity components in both weight matrices. They intend to avoid unstable behavior due to high angular velocity. In this work they were set to 40.0.
- $R_y$and $R_z$ comprise linearly decreasing weights to reduce abrupt accelerations caused by the forces. This weight starts at 0.100 at time step $t_0$ and decreases to 0.050 at the terminal step, allowing higher support when approaching the target  [44].
- $Q_{target}$ is a weight that influences the deviations from the target at the collision time. This corresponds with the first row in the cost function $h_{t_0+N}$. A high value (800) is assigned only in $W_{t_0+N}$ to penalize such deviations. Since the positions in the *z*-axis are not required, the second element of the diagonal remains zero.

## Appendix C Questionnaires

### C. 1 Locus of Control (LOC)

The LOC questionnaire  [34] is formed by 23 multiple-choice questions and 6 trap questions. Scores can be found at the end of the list. A high score means *External LOC*, and a low score means *Internal LOC*.

#### C.1.1 For each question, select the statement that you agree with the most

1. *a.* Children get into trouble because their parents punish them too much.
  *b.* The trouble with most children nowadays is that their parents are too easy with them.
2. *a.* Many of the unhappy things in people’s lives are partly due to bad luck.
  *b.* People’s misfortunes result from the mistakes they make.
3. *a.* One of the major reasons why we have wars is because people don’t take enough interest in politics.
  *b.* There will always be wars, no matter how hard people try to prevent them.
4. *a.* In the long run, people get the respect they deserve in this world.
  *b.* Unfortunately, an individual’s worth often passes unrecognized, no matter how hard he tries.
5. *a.* The idea that teachers are unfair to students is nonsense.
  *b.* Most students don’t realize the extent to which their grades are influenced by accidental happening.
6. *a.* Without the right breaks, one cannot be an effective leader.
  *b.* Capable people who fail to become leaders have not taken advantage of their opportunities.
7. *a.* No matter how hard you try, some people just don’t like you.
  *b.* People who can’t get others to like them don’t understand how to get along with others.
8. *a.* Heredity plays a major role in determining one’s personality.
  *b.* It is one’s experiences in life which determine what they’re like.
9. *a.* I have often found that what is going to happen will happen.
  *b.* Trusting fate has never turned out as well for me as making a decision to take a definite course of action.
10. *a.* In the case of the well-prepared student, there is rarely, if ever, such a thing as an unfair test.
  *b.* Many times, exam questions tend to be so unrelated to coursework that studying is really useless.
11. *a.* Becoming a success is a matter of hard work; luck has little or nothing to do with it.
  *b.* Getting a good job depends mainly on being in the right place at the right time.
12. *a.* The average citizen can have an influence on government decisions.
  *b.* This world is run by the few people in power, and there is not much the little guy can do about it.
13. *a.* When I make plans, I am almost certain that I can make them work.
  *b.* It is not always wise to plan too far ahead because many things turn out to be a matter of good or bad fortune anyhow.
14. *a.* There are certain people who are just no good.
  *b.* There is some good in everybody
15. *a.* In my case, getting what I want has little or nothing to do with luck.
  *b.* Many times, we might just as well decide what to do by flipping a coin.
16. *a.* Who gets to be the boss often depends on who was lucky enough to be in the right place first.
  *b.* Getting people to do the right thing depends upon ability. Luck has little or nothing to do with it.
17. *a.* As far as world affairs are concerned, most of us are the victims of forces we can neither understand nor control.
  *b.* By taking an active part in political and social affairs, the people can control world events.
18. *a.* Most people don’t realize the extent to which their lives are controlled by accidental happenings.
  *b.* There really is no such thing as ’luck.’
19. *a.* One should always be willing to admit mistakes.
  *b.* It is usually best to cover up one’s mistakes.
20. *a.* It is hard to know whether or not a person really likes you.
  *b.* How many friends you have depends upon how nice a person you are.
21. *a.* In the long run, the bad things that happen to us are balanced by the good ones.
  *b.* Most misfortunes are the result of lack of ability, ignorance, laziness, or all three.
22. *a.* With enough effort, we can wipe out political corruption.
  *b.* It is difficult for people to have much control over the things politicians do in office.
23. *a.* Sometimes, I can’t understand how teachers arrive at the grades they give.
  *b.* There is a direct connection between how hard I study and the grades I get.
24. *a.* A good leader expects people to decide for themselves what they should do.
  *b.* A good leader makes it clear to everybody what their jobs are.
25. *a.* Many times, I feel that I have little influence over the things that happen to me.
  *b.* It is impossible for me to believe that chance or luck plays an important role in my life.
26. *a.* People are lonely because they don’t try to be friendly.
  *b.* There’s not much use in trying too hard to please people; if they like you, they like you.
27. *a.* There is too much emphasis on athletics in high school.
  *b.* Team sports are an excellent way to build character.
28. *a.* What happens to me is my own doing.
  *b.* Sometimes, I feel that I don’t have enough control over the direction my life is taking.
29. *a.* Most of the time I can’t understand why politicians behave the way they do.
  *b.* In the long run, the people are responsible for bad government on a national as well as on a local level.

**Score one point for each of the following.**

2.a, 3.b, 4.b, 5.b, 6.a, 7.a, 9.a, 10.b, 11.b, 12.b, 13.b, 15.b, 16.a, 17.a, 18.a, 20.a, 21. a, 22.b, 23.a, 25.a, 26.b, 28.b, 29.a.

### C.2 Autotelic personality

The subscales Transform of Challenge and Transform of Boredom from  [33] are based on a seven-point-based questionnaire. The scale depends on whether they agree (7) or not (1) with the statement. The higher the score, the higher the level of this trait.

- **Transform of Challenge**
  I like solving complex problems.
  I enjoy playing difficult games.
  I would prefer a job that is challenging over a job that is easy.
- **Transform of Boredom**
  Repetitive tasks are enjoyable.
  I have fun doing things that others say are boring.
  I am able to find pleasure even in routine types of work.
  I make a game out of chores.

### C.3 Gaming style

The subscales Achiever and Free Spirit from  [39] are based on a seven-point-based questionnaire. The scale depends on whether they agree (7) or not (1) with the statement. The higher the score, the more they fit in this subcategory.

- **Achiever**
  I like mastering difficult tasks.
  It is important to me to always carry out my tasks completely.
  I like defeating obstacles.
  I am very ambitious.
  It is difficult for me to let go of a problem before I have found a solution.
- **Free Spirit**
  Being independent is important to me.
  It is important to me to follow my own path.
  I often let my curiosity guide me.
  I like to try new things.
  I prefer setting my own goals.

### C.4 Human-robot interaction questions

The questions regarding human-robot interaction (HRI-questions) are three questions specifically created for this experiment. They aimed to reflect the subjective experience of the users who underwent the guidance approach. Those questions are based on a seven-point-based questionnaire.

- Was the robot interaction disturbing or helpful?
  Disturbing (-3) to helpful (3)
- Was the robot interaction restricting or permitting your own movements?
  Restricting (-3) to permitting (3)
- The robot interaction frustrated me.
  Not true at all (1) to very true (7)

## Appendix D Model selection

### D.1 Models evaluated to infer motor learning related to error reduction M1.1

Several candidate models were evaluated that differed in the inclusion of interaction terms, fixed effects, and trait combinations. All models employed the same dataset as described in the main manuscript and were systematically reduced based on the Akaike Information Criterion (AIC) and Bayesian Information Criterion (BIC) reported in Table 4. The acronyms employed in the models correspond to the following variables: $AC_{c}$: Achiever gaming style centered, $FS_{c}$: Free Spirit gaming style centered, $TC_{c}$: Transform of Challenge centered, $TB_{c}$: Transform of Boredom centered, $LOC_{c}$: Locus of Control centered, *ID*: Personal identifier, *wIndex*: Wall index within the set, *NewWalls*: walls corresponding to the position transfer task, *sIndex*: Set index within the stage.

Model 1 (employed in Appendix A):

$$\begin{aligned} \log _{{10}} |{\text{Error}}| & = (AC_{c} + FS_{c} + TC_{c} + TB_{c} + LOC_{c} ) \\ & \times Stage + (TC_{c} + LOC) \times Stage \\ & \times Group + (1|ID) + (1|wIndex), \\ \end{aligned} $$

Model 2:

$$ \begin{aligned} \log _{{10}} |{\text{Error}}| & = (AC_{c} + FS_{c} + TC_{c} + TB_{c} + LOC_{c} ){\mkern 1mu} \\ {\mkern 1mu} & \times (Group \times Task \times Stage) \\ & + (1|ID){\mkern 1mu} {\mkern 1mu} {\mkern 1mu} + (1|wIndex:NewWalls), \\ \end{aligned} $$

Model 3:

$$ \begin{aligned} \log _{{10}} |{\text{Error}}| & = (AC_{c} + FS_{c} + TC_{c} + TB_{c} + LOC_{c} ){\mkern 1mu} \\ & \times (Group + Task \times Stage) + (1|ID) \\ & + (1|wIndex:NewWalls), \\ \end{aligned}$$

Model 4:

$$ \begin{aligned} \log _{{10}} |{\text{Error}}| & = (AC_{c} + FS_{c} + TC_{c} + TB_{c} + LOC_{c} ) \\ & \times (Group + Task + Stage){\mkern 1mu} {\mkern 1mu} {\mkern 1mu} + Task \\ & \times Stage + (1|ID){\mkern 1mu} {\mkern 1mu} \\ & + (1|wIndex:NewWalls), \\ \end{aligned} $$

Model 5:

$$\begin{aligned} \log _{{10}} |{\text{Error}}| & = (AC_{c} + FS_{c} + TC_{c} + TB_{c} + LOC_{c} ) \\ {\mkern 1mu} {\mkern 1mu} {\mkern 1mu} & \times (Group + Task + Stage + sIndex) \\ & + Task \times Stage \times sIndex {\mkern 1mu} \\ & + (1|ID) + (1|wIndex:NewWalls), \\ \end{aligned} $$

Model 6:

$$\begin{aligned} \log _{10}| \text {Error}| = \, &({TC}_{c} +{LOC_c}) \, \\ &\times (Group+ Task+ Stage+sIndex)\\ &+ Task\times Stage\times sIndex\\ &+ (1|ID) \, + (1|{wIndex}:{NewWalls}), \end{aligned}$$

Model 7:

$$\begin{aligned} \log _{{10}} |{\text{Error}}| & = (AC_{c} + TB_{c} ){\mkern 1mu} {\mkern 1mu} {\mkern 1mu} {\mkern 1mu} {\mkern 1mu} {\mkern 1mu} {\mkern 1mu} {\mkern 1mu} {\mkern 1mu} {\mkern 1mu} {\mkern 1mu} {\mkern 1mu} {\mkern 1mu} {\mkern 1mu} {\mkern 1mu} {\mkern 1mu} {\mkern 1mu} {\mkern 1mu} {\mkern 1mu} {\mkern 1mu} {\mkern 1mu} {\mkern 1mu} {\mkern 1mu} {\mkern 1mu} {\mkern 1mu} {\mkern 1mu} {\mkern 1mu} {\mkern 1mu} {\mkern 1mu} {\mkern 1mu} {\mkern 1mu} \\ & \times (Group + Task + Stage + sIndex) \\ {\mkern 1mu} & + Task \times Stage \times sIndex {\mkern 1mu} \\ & + (1|ID) + (1|wIndex:NewWalls), \\ \end{aligned} $$

Model 8:

$$ \begin{aligned} \log _{{10}} |{\text{Error}}| & = Group \times (Task + Stage) \\ & + (TC_{c} + LOC_{c} ) \\ & \times (Task + Stage + sIndex) \\ & + Task \times Stage + Group \\ & \times TC_{c} \times Stage + (1|ID) \\ & + (1|wIndex:NewWalls), \\ \end{aligned} $$

We compared models 1 to 8 with AIC and BIC. Results can be found in Table 4.

**Table 4:** AIC and BIC values from models 1 to 8. These values were employed to select the best candidate to be the model M1.1

Model	AIC	BIC
1	19148.19	19375.44
2	19063.12	19903.94
3	18991.35	19468.57
4	18941.24	19274.54
5	18995.21	19426.98
6	18928.02	19200.72
7	18928.80	19201.50
8	18916.06	19166.04

Model 8 was selected as the final model, as it exhibited the lowest AIC and BIC values among all candidates, indicating the best trade-off between goodness-of-fit and model complexity. Notably, traits excluded from the final model (i.e., those not retained in Model 8) did not exhibit statistically significant effects in any of the candidate models, therefore hypothesis H1.3 was not supported by the data.

### D.2 Models proposed for the M1.2, related to human-robot interaction

To maintain consistency with our modeling approach, facilitate parallel interpretation of effects in the main document in this paper, and streamline the analysis, we began the construction of Model M1.2 using the selected model to evaluate M1.1. However, inspired by the results in [43] related to the training phase of the experiment, an extra term was added for the interaction between LOC, group, and stage. Furthermore, we acknowledge that the previously excluded personality traits might lead to new outcomes due to the use of a different dependent variable ($\log _{10}|IntForce|$). Therefore, we introduced a third model (Model 11) that included the full set of traits for completeness.

Model 9:

$$ \begin{aligned} \log _{{10}} |{\text{intForce}}| & = Group \times (Task + Stage) \\ & + (TC_{c} + LOC_{c} ) \\ & \times (Task + Stage + sIndex) \\ & + Task \times Stage + Group \times TC_{c} \\ & \times Stage + (1|ID) \\ & + (1|wIndex:NewWalls). \\ \end{aligned} $$

Model 10:

$$ \begin{aligned} \log _{{10}} |{\text{intForce}}| & = Group \times (Task + Stage) \\ & + (TC_{c} + LOC_{c} ) \\ & \times (Task + Stage + sIndex) \\ & + Task \times Stage + Group \times TC_{c} \\ & \times Stage + (1|ID) \\ & + (1|wIndex:NewWalls). \\ \end{aligned} $$

Model 11:

$$\begin{aligned} \log _{{10}} |{\text{intForce}}| & = Group \times (Task + Stage) \\ & + (AC_{c} + FS_{c} + TB_{c} + TC_{c} + LOC_{c} ) \\ & \times (Task + Stage + sIndex) \\ & + Task \times Stage + Group \times TC_{c} \\ & \times Stage + Group \times LOC_{c} \times Stage \\ & + (1|ID) + (1|wIndex:NewWalls). \\ \end{aligned} $$

We compared models 9 to 11 with AIC and BIC. Results can be found in Table 5.

**Table 5:** AIC and BIC values from models 9 to 11. These values were employed to select the best candidate to be the model M1.2

Model	AIC	BIC
9	– 2957.828	– 2707.853
10	– 2961.459	– 2688.759
11	– 2982.459	– 2573.410

As AIC and BIC favored different models, we chose to retain Model 10 for the main analysis and to show in the main manuscript. This decision preserves a consistent results structure across sections while acknowledging the relevance of the added LOC-group-stage interaction, which showed statistical significance and aligns with prior findings [43].

However, Model 11 revealed a significant interaction between the other traits—-i.e., Transform of Boredom, Free Spirit, and Achiever gaming styles—-and the interaction force during both short- and long-term retention in the control group. This finding would support Hypothesis H1.4. Yet, fully exploring these additional effects would require extensive model expansion and iteration, along with the presentation of a broader set of results. Additionally, the inclusion of a large number of variables and interaction terms in Model 11 raises the possibility that some low *p*-values may reflect statistical instability or overfitting rather than true effects, which should be addressed carefully. To maintain the focus and scope of this paper, we reserve this analysis for future work. For completeness, the full results of Model 11 are provided in Appendix E

### D.3 Models proposed for the human-robot interaction perception analysis, M2

The development of Model M2 followed the procedure described in the Human-robot interaction perception model (M2) Section. However, given the low statistical significance of the coefficients associated with the Transform of Boredom trait, we explored whether excluding this variable would improve model performance. To this end, we compared two versions of the model: one including all traits, and another omitting Transform of Boredom.

Model 12:

$$ \begin{aligned} \log _{{10}} |{\text{Error}}| & = {\mkern 1mu} (AC_{c} + FS_{c} + TB_{c} + TC_{c} + LOC_{c} ){\mkern 1mu} \\ & \times HRIQuestion \times Stage \\ & + (1|ID) + (1|wIndex), \\ \end{aligned} $$

Model 13:

$$ \begin{aligned} \log _{{10}} |{\text{Error}}| & = (AC_{c} + FS_{c} + TC_{c} + LOC_{c} ){\mkern 1mu} \\ & \times HRIQuestion \times Stage{\mkern 1mu} \\ & + (1|ID) + (1|wIndex), \\ \end{aligned} $$

The results of the AIC and BIC are shown in Table 6.

**Table 6:** AIC and BIC values from models 12 to 13. These values were employed to select the best candidate to be the model M2

Model	AIC	BIC
12	20284.14	20670.46
13	20274.72	20600.44

Model 13 yielded lower AIC and BIC values, indicating improved model fit without the inclusion of the Transform of Boredom trait. As this variable did not contribute meaningful explanatory power in this context, Model 11 was selected for the final analysis.

## Appendix E Linear Mixed Models output

See Tables 7, 8, 9 and 10.

**Table 7:** Results from the LMM identified as M1.1

Variable	Estimate ($\beta$)	Std. Error	t value	*p*-value	Corr. *p*-value
**(Intercept)**	– 1.275	3.461e-02	-36.820	$<$ 2e-16	**9.495e-57*****
**Group E**	– 4.214e-02	3.714e-02	– 1.135	2.618e-01	1
**t1**	3.913e-02	3.503e-02	1.117	2.681e-01	1
**t2**	– 8.029e-02	1.887e-02	– 4.255	2.100e-05	**6.303e-04*****
**STR**	– 2.053e-01	1.897e-02	– 10.820	$<$ 2e-16	**1.031e-25*****
**LTR**	– 2.089e-01	1.897e-02	– 11.020	$<$ 2e-16	**1.255e-26*****
$\mathbf {TC_c}$	– 8.841e-02	2.603e-01	– 0.340	7.356e-01	1
$\mathbf {LOC_c}$	3.781e-02	5.502e-02	0.687	4.948e-01	1
**Set Index**	– 2.630e-02	7.668e-03	– 3.430	6.050e-04	**1.814e-02***
**Group E - t1**	2.576e-02	1.912e-02	1.347	1.779e-01	1
**Group E - t2**	1.119e-03	1.912e-02	0.059	9.533e-01	1
**Group E - STR**	6.015e-02	1.914e-02	3.143	1.677e-03	**5.031e-02**^**·**^
**Group E - LTR**	4.445e-02	1.914e-02	2.322	2.022e-02	6.067e-01
**t1 - **$\mathbf {TC_c}$	– 6.802e-03	8.063e-02	– 0.084	9.328e-01	1
**t2 - **$\mathbf {TC_c}$	2.549e-01	8.063e-02	3.161	1.573e-03	**4.719e-02***
**STR -** $\mathbf {TC_c}$	– 3.857e-01	1.371e-01	– 2.813	4.913e-03	1.473951e-01
**LTR - **$\mathbf {TC_c}$	– 2.711e-01	1.371e-01	-1.977	4.803e-02	1
$\mathbf {TC_c}$ **- Set Index**	6.474e-02	6.496e-02	0.997	3.189e-01	1
**t1 - **$\mathbf {LOC_c}$	– 2.798e-02	2.770e-02	– 1.010	3.125e-01	1
**t2 -** $\mathbf {LOC_c}$	-1.707e-02	2.770e-02	– 0.616	5.378e-01	1
**STR - **$\mathbf {LOC_c}$	4.206e-02	2.776e-02	1.515	1.298e-01	1
**LTR - **$\mathbf {LOC_c}$	7.228e-02	2.776e-02	2.603	9.237e-03	2.771e-01
$\mathbf {LOC_c}$**- Set Index**	-6.083e-03	2.247e-02	-0.271	7.867e-01	1
**t1 - STR**	-5.329e-02	2.300e-02	-2.317	2.053e-02	6.159e-01
**t2 - STR**	6.582e-02	2.300e-02	2.862	4.222e-03	1.267e-01
**t1 - LTR**	-6.801e-03	2.300e-02	-0.296	7.675e-01	1
**t2 - LTR**	5.093e-02	2.300e-02	2.214	2.684e-02	8.051e-01
**Group E - **$\mathbf {TC_c}$	-1.186e-01	3.133e-01	-0.378	7.070e-01	1
**Group E - STR - **$\mathbf {TC_c}$	3.809e-01	1.691e-01	2.252e	2.431e-02	7.293e-01
**Group E - LTR -** $\mathbf {TC_c}$	2.255e-01	1.691e-01	1.334	1.823e-01	1

Values in bold indicate statistically significant results (*p* < 0.05). Near-significant *p*-values are also shown in bold but marked with a dot (·) instead of an asterisk (*)

*($p < 0.05$), **($p < 0.01$), ***($p < 0.001$). Std.: Standard; Corr.: Corrected; E: Experimental; t1: Position transfer task; t2: Dynamics transfer task; STR: Short-term retention stage; LTR: Long-term retention stage; *TC*: Transform of Challenge; *LOC*: Locus of Control. Lowercase ’c’ denotes centering the questionnaire results on the mean

**Table 8:** Results from the LMM identified as M1.2.

Variable	Estimate ($\beta$)	Std. Error	t value	*p*-value	Corr. *p*-value
**(Intercept)**	– 2.937e-01	3.765e-02	– 7.802	1.47e-10	**4.842e-09*****
**Group E**	2.711e-03	2.374e-02	0.114	9.096e-01	1
**t1**	– 5.991e-02	4.808e-02	– 1.246	2.200e-01	1
**t2**	– 3.475e-03	8.772e-03	– 0.396	6.920e-01	1
**STR**	– 8.802e-02	8.836e-03	– 9.962	$<$ 2e-16	**8.826e-22*****
**LTR**	-7.716e-02	8.836e-03	-8.732	$<$ 2e-16	**9.196e-17*****
$\mathbf {TC_c}$	5.077e-02	1.682e-01	0.302	7.644e-01	1
$\mathbf {LOC_c}$	– 2.009e-02	5.566e-02	– 0.361	7.201e-01	1
**Set Index**	2.489e-02	3.565e-03	6.982	3.033e-12	**1.001e-10*****
**Group E - t1**	1.813e-02	8.890e-03	2.039	4.142e-02	1
**Group E - t2**	4.954e-03	8.890e-03	0.557	5.774e-01	1
**Group E - STR**	1.815e-02	8.900e-03	2.039	4.146e-02	1
**Group E - LTR**	– 1.684e-04	8.900e-03	-0.019	9.849e-01	1
**t1 -** $\mathbf {TC_c}$	1.071e-02	3.749e-02	0.286	7.750e-01	1
**t2 - **$\mathbf {TC_c}$	6.570e-02	3.749e-02	1.753	7.967e-02	1
**STR - **$\mathbf {TC_c}$	– 3.569e-01	6.378e-02	– 5.597	2.225e-08	**7.342e-07*****
**LTR -** $\mathbf {TC_c}$	– 3.515e-02	6.378e-02	– 0.551	5.815e-01	1
$\mathbf {TC_c}$**- Index**	5.775e-02	3.020e-02	1.912	5.584e-02	1
**t1 - **$\mathbf {LOC_c}$	– 7.242e-03	1.288e-02	– 0.562	5.739e-01	1
**t2 - **$\mathbf {LOC_c}$	5.274e-03	1.288e-02	0.409	6.822e-01	1
**STR - **$\mathbf {LOC_c}$	– 4.314e-03	2.108e-02	– 0.205	8.379e-01	1
**LTR - **$\mathbf {LOC_c}$	– 2.250e-03	2.108e-02	– 0.107	9.150e-01	1
$\mathbf {LOC_c}$**- Set Index**	– 1.189e-02	1.045e-02	-1.138	2.549e-01	1
**t1 - STR**	3.986e-02	1.069e-02	3.727	1.945e-04	**6.417e-03****
**t2 - STR**	– 8.457e-03	1.069e-02	– 0.791	4.291e-01	1
**t1 - LTR**	5.991e-02	1.069e-02	5.602	2.159e-08	**7.124e-07*****
**t2 - LTR**	– 2.000e-03	1.069e-02	-0.187	8.517e-01	1
**Group E - **$\mathbf {TC_c}$	– 3.073e-01	2.056e-01	– .495	1.434e-01	1
**Group E - **$\mathbf {LOC_c}$	– 2.149e-02	6.945e-02	– 0.309	7.587e-01	1
**Group E - STR -** $\mathbf {TC_c}$	5.197e-01	7.893e-02	6.584	4.732e-11	**1.562e-09*****
**Group E - LTR - **$\mathbf {TC_c}$	8.129e-02	7.893e-02	1.030	3.031e-01	1
**Group E - STR -** $\mathbf {LOC_c}$	8.337e-02	2.666e-02	3.127	1.771e-03	**5.843e-02**^**·**^
**Group E - LTR -** $\mathbf {LOC_c}$	1.281e-01	2.666e-02	4.805	1.567e-06	**5.170e-05*****

Values in bold indicate statistically significant results (*p* < 0.05). Near-significant *p*-values are also shown in bold but marked with a dot (·) instead of an asterisk (*)

*($p < 0.05$), **($p < 0.01$), ***($p < 0.001$). Std.: Standard; Corr.: Corrected; E: Experimental; t1: Position transfer task; t2: Dynamics transfer task; STR: Short-term retention stage; LTR: Long-term retention stage; *TC*: Transform of Challenge; *LOC*: Locus of Control. Lowercase ’c’ denotes centering the questionnaire results on the mean

**Table 9:** Results from the alternative LMM showed in Appendix D and candidate for M1.2

Variable	Estimate ($\beta$)	Std. Error	t value	*p*-value	Corr. *p*-value
**(Intercept)**	– 2.923e-01	3.765e-02	– 7.763	1.75e-10	**8.944e-09*****
**STR**	– 8.907e-02	8.817e-03	– 10.102	$<$ 2e-16	**3.322e-22*****
**LTR**	– 7.845e-02	8.817e-03	– 8.897	$<$ 2e-16	**3.262e-17*****
$\mathbf {FS_c}$	– 3.691e-01	1.980e-01	– 1.864	0.069757	1
$\mathbf {TB_c}$	– 2.523e-01	8.322e-02	– 3.031	0.004295	2.190e-01
**Set Index**	2.489e-02	3.551e-03	7.010	2.49e-12	**1.272e-10*****
**Group E - t1**	2.036e-02	8.926e-03	2.280	0.022597	1
**Group E - STR**	2.238e-02	8.932e-03	2.505	0.012255	6.250e-01
**STR - **$\mathbf {TC_c}$	– 3.415e-01	7.853e-02	– 4.349	1.38e-05	**7.027e-04*****
**LTR - **$\mathbf {LOC_c}$	– 5.536e-02	2.279e-02	– 2.429	0.015133	7.718e-01
$\mathbf {LOC_c}$**- Set Index**	– 2.003e-02	1.137e-02	– 1.762	0.078132	1
**t1 - **$\mathbf {FS_c}$	1.450e-01	7.264e-02	1.997	0.045847	1
**t2 - **$\mathbf {FS_c}$	1.509e-01	7.264e-02	2.078	0.037767	1
**STR - **$\mathbf {FS_c}$	3.154e-01	7.335e-02	4.300	1.72e-05	**8.762e-04*****
**LTR - **$\mathbf {FS_c}$	4.124e-01	7.335e-02	5.622	1.92e-08	**9.814e-07*****
$\mathbf {FS_c}$**- Index**	5.459e-02	5.884e-02	0.928	0.353471	1
**t1 - **$\mathbf {TB_c}$	6.351e-02	3.026e-02	2.099	0.035834	1
**t2 - **$\mathbf {TB_c}$	1.275e-02	3.026e-02	0.421	0.673420	1
**STR - **$\mathbf {TB_c}$	9.963e-02	3.085e-02	3.230	0.001242	6.333e-02
**LTR - **$\mathbf {TB_c}$	2.054e-01	3.085e-02	6.658	2.87e-11	**1.466e-09*****
$\mathbf {TB_c}$**- Set Index**	4.864e-02	2.470e-02	1.969	0.048972	1
**t1 - **$\mathbf {AC_c}$	– 4.531e-02	5.044e-02	– 0.898	0.369039	1
**t2 - **$\mathbf {AC_c}$	– 7.972e-02	5.044e-02	– 1.580	0.114015	1
**STR - **$\mathbf {AC_c}$	– 2.888e-01	5.081e-02	– 5.684	1.34e-08	**6.853e-07*****
**LTR - **$\mathbf {AC_c}$	– 2.654e-01	5.081e-02	– 5.222	1.79e-07	**9.133e-06*****
$\mathbf {AC_c}$ **- Index**	– 2.448e-02	4.116e-02	– 0.595	0.552073	1
**t1 - STR**	3.986e-02	1.065e-02	3.742	0.000183	**9.355e-03****
**t2 - STR**	– 8.457e-03	1.065e-02	– 0.794	0.427296	1
**t1 - LTR**	5.991e-02	1.065e-02	5.624	1.90e-08	**9.691e-07*****
**t2 - LTR**	– 2.000e-03	1.065e-02	– 0.188	0.851097	1
**Group E - **$\mathbf {TC_c}$	– 3.642e-01	2.070e-01	– 1.760	0.087370	1
**Group E - **$\mathbf {LOC_c}$	-5.099e-02	7.109e-02	– 0.717	0.478027	1
**Group E - STR - **$\mathbf {TC_c}$	5.913e-01	7.939e-02	7.448	1.00e-13	**5.105e-12*****
**Group E - LTR - **$\mathbf {TC_c}$	1.758e-01	7.939e-02	2.214	0.026813	1
**Group E - STR - **$\mathbf {LOC_c}$	1.099e-01	2.727e-02	4.030	5.60e-05	**2.854e-03****
**Group E - LTR - **$\mathbf {LOC_c}$	1.681e-01	2.727e-02	6.165	7.25e-10	**3.698e-08*****

Values in bold indicate statistically significant results (*p* < 0.05)

Only significative values are shown to reduce table space. *($p < 0.05$), **($p < 0.01$), ***($p < 0.001$). Std.: Standard; Corr.: Corrected; E: Experimental; t1: Position transfer task; t2: Dynamics transfer task; STR: Short-term retention stage; LTR: Long-term retention stage; *TC*: Transform of Challenge; *LOC*: Locus of Control, $AC_c$: Achiever Gaming Style, $FS_c$: Free Spirit Gaming Style, $TB_c$: Transform of Boredom. Lowercase ’c’ denotes centering the questionnaire results on the mean

**Table 10:** Results from the LMM identified as M2

Variable	Estimate ($\beta$)	Std. Error	t value	*p*-value	Corr. *p*-value
**(Intercept)**	– 1.323	5.917e-02	– 22.364	9.778e-16	**3.911e-14*****
$\mathbf {LOC_c}$	– 7.169e-02	1.537e-01	– 0.466	6.482e-01	1
$\mathbf {FS_c}$	– 6.318e-01	7.256e-01	– 0.871	3.988e-01	1
$\mathbf {AC_c}$	– 8.718e-02	5.300e-01	– 0.164	8.717e-01	1
$\mathbf {TC_c}$	2.382e-01	7.356e-01	0.324	7.509e-01	1
**Frustration**	– 4.402e-04	4.809e-02	– 0.009	9.928e-01	1
**TR**	– 1.526e-01	2.191e-02	– 6.964	3.442e-12	**1.377e-10*****
**STR**	– 1.608e-01	3.001e-02	– 5.360	8.445e-08	**3.378e-06*****
**LTR**	– 1.601e-01	3.001e-02	– 5.334	9.746e-08	**3.898e-06*****
$\mathbf {LOC_c}$**- Frustration**	– 2.807e-02	1.065e-01	– 0.264	7.960e-01	1
$\mathbf {FS_c}$**- Frustration**	– 9.047e-01	9.108e-01	– 0.993	3.376e-01	1
$\mathbf {AC_c}$**- Frustration**	1.569e-04	6.348e-01	0.000	9.998e-01	1
$\mathbf {TC_c}$**- Frustration**	2.874e-01	9.365e-01	0.307	7.635e-01	1
$\mathbf {LOC_c}$**- TR**	8.537e-03	6.323e-02	0.135	8.926e-01	1
$\mathbf {LOC_c}$**- STR**	5.658e-02	8.658e-02	0.653	5.134e-01	1
$\mathbf {LOC_c}$**- LTR**	1.919e-01	8.658e-02	2.217	2.666e-02	1
$\mathbf {FS_c}$**- TR**	2.352e-0	2.985e-01	0.788	4.307e-01	1
$\mathbf {FS_c}$**- STR**	– 4.773e-01	4.087e-01	– 1.168	2.430e-01	1
$\mathbf {FS_c}$**- LTR**	1.747e-01	4.087e-01	0.427	6.690e-01	1
$\mathbf {AC_c}$**- TR**	3.797e-02	2.180e-01	0.174	8.617e-01	1
$\mathbf {AC_c}$**- STR**	– 2.251e-01	2.986e-01	– 0.754	4.510e-01	1
$\mathbf {AC_c}$**- LTR**	– 3.805e-01	2.986e-01	– 1.275	2.025e-01	1
$\mathbf {TC_c}$**- TR**	1.661e-01	3.026e-01	0.549	5.831e-01	1
$\mathbf {TC_c}$**- STR**	3.439e-01	4.144e-01	0.830	4.066e-01	1
$\mathbf {TC_c}$**- LTR**	– 1.263e-01	4.144e-01	-0.305	7.606e-01	1
**Frustration - TR**	4.954e-02	1.978e-02	2.504	1.228e-02	4.913e-01
**Frustration - STR**	2.582e-02	2.709e-02	0.953	3.404e-01	1
**Frustration - LTR**	5.097e-02	2.709e-02	1.882	5.992e-02	1
$\mathbf {LOC_c}$**- Frustration - TR**	– 2.698e-02	4.382e-02	– 0.616	5.380e-01	1
$\mathbf {LOC_c}$**- Frustration - STR**	– 1.027e-02	6.000e-02	– 0.171	8.641e-01	1
$\mathbf {LOC_c}$**- Frustration - LTR**	– 3.728e-02	6.000e-02	– 0.621	5.343e-01	1
$\mathbf {FS_c}$**- Frustration - TR**	7.608e-01	3.747e-01	2.031	4.232e-02	1
$\mathbf {FS_c}$**- Frustration - STR**	6.014e-01	5.131e-01	1.172	2.411e-01	1
$\mathbf {FS_c}$**- Frustration - LTR**	1.951	5.131e-01	3.802	1.443e-04	**5.772e-03****
$\mathbf {AC_c}$**- Frustration - TR**	1.151e-01	2.611e-01	0.441	6.594e-01	1
$\mathbf {AC_c}$**- Frustration - STR**	– 7.836e-04	3.576e-01	– 0.002	9.982e-01	1
$\mathbf {AC_c}$**- Frustration - LTR**	3.753e-01	3.576e-01	1.050	2.939e-01	1
$\mathbf {TC_c}$**- Frustration - TR**	– 2.339e-01	3.853e-01	– 0.607	5.438e-01	1
$\mathbf {TC_c}$**- Frustration - STR**	8.707e-02	5.275e-01	0.165	8.689e-01	1
$\mathbf {TC_c}$**- Frustration - LTR**	– 1.326	5.275e-01	– 2.514	1.195e-02	4.780e-01

Values in bold indicate statistically significant results (*p* < 0.05)

*($p < 0.05$), **($p < 0.01$), ***($p < 0.001$). Std.: Standard; Corr.: Corrected; TR: Training stage; STR: Short-term retention stage; LTR: Long-term retention stage; *TC*: Transform of Challenge; *LOC*: Locus of Control; *FS*: Free Spirit; *AC*: Achiever. Lowercase ’c’ denotes centering the questionnaire results on the mean
